# sEH inhibition attenuates mtROS-mediated NLRP3 inflammasome activation by promoting mitophagy in tubular epithelial cells in diabetic kidney disease

**DOI:** 10.3389/fimmu.2026.1767802

**Published:** 2026-04-28

**Authors:** Xushun Jiang, Wenge Huang, Wen Wen, Ting Liu, Wei Ren, Jia Shi, Junling He, Xiaogang Du

**Affiliations:** 1 Health Management Center, The First Affiliated Hospital of Chongqing Medical University, Chongqing, China; 2 Metabolism and Immunology Laboratory for Urological Diseases, The First Affiliated Hospital of Chongqing Medical University, Chongqing, China; 3 Department of Nephrology, Metabolism and Immunology Laboratory for Urological Diseases, The First Affiliated Hospital of Chongqing Medical University, Chongqing, China; 4 Department of Cardiology, The First Affiliated Hospital of Chongqing Medical University, Chongqing, China; 5 Department of Endocrinology, The First Affiliated Hospital of Chongqing Medical University, Chongqing, China

**Keywords:** diabetic kidney disease, mitochondrial reactive oxygen species, mitophagy, NLRP3 inflammasome, soluble epoxide hydrolase

## Abstract

Inflammation is a key driver of diabetic kidney disease (DKD) progression, with the NOD-like receptor family pyrin domain-containing 3 (NLRP3) inflammasome representing a promising therapeutic target. Soluble epoxide hydrolase (sEH), an enzyme that inactivates renoprotective epoxyeicosatrienoic acids into less active diols, has been implicated in renal pathophysiology. This study investigated the role of sEH in renal tubular NLRP3 inflammasome activation and its underlying mechanisms in DKD. We observed upregulated sEH expression in high glucose (HG)-stimulated human proximal tubular epithelial (HK-2) cells and diabetic kidneys. Pharmacological inhibition of sEH attenuated diabetes-induced mitochondrial damage, mitochondrial reactive oxygen species (mtROS) production, NLRP3 inflammasome activation, and renal dysfunction both *in vivo* and *in vitro*. Furthermore, sEH inhibition restored autophagy flux and enhanced PINK1/Parkin mediated-mitophagy. Activation of mitophagy by the autophagy inducer rapamycin (Rapa) alleviated HG-induced mitochondrial impairment, mtROS overproduction, and NLRP3 inflammasome activation in HK-2 cells, suggesting that the anti-inflammatory effect of sEH inhibition is mediated through mitophagy regulation. Further mechanistic studies indicated that sEH inhibition promotes mitophagy, thereby improving mitochondrial function, reducing mtROS generation, and subsequently suppressing mtROS-dependent NLRP3 inflammasome activation in DKD. In conclusion, our findings establish a novel link between sEH and NLRP3 inflammasome activation in DKD pathogenesis, highlighting sEH inhibition as a promising therapeutic strategy for DKD treatment.

## 1 Introduction

Diabetic kidney disease (DKD) is a recognized microvascular complication in patients with diabetes that ultimately results in progressive kidney failure. Globally, it is estimated to affect nearly half a billion people, with prevalence projected to increase by 25% by 2030 and by 51% by 2045, posing substantial challenges to healthcare systems worldwide (1). Emerging evidence indicates that renal tubular injury may occur earlier than glomerular injury. Therefore, the emergence of a “tubulocentric view of DKD” has drawn increasing attention to the critical role played by tubular epithelial cells (TECs) dysfunction in the pathogenesis of DKD (2, 3).

The pathogenesis of DKD involves a complex, multifactorial process mediated by numerous signaling pathways and molecular factors, including hemodynamic factors, metabolic alterations, oxidative stress, inflammation, and so on (4). Among these, inflammation serves as a key driver of DKD progression. In addition to classical pathogenic stimuli, sterile triggers such as hyperglycemia, dyslipidemia, and ischemia can initiate renal inflammatory responses (5). The NOD-like receptor protein 3 (NLRP3) inflammasome—a cytosolic multiprotein complex composed of NLRP3, ASC (apoptosis-associated speck-like protein), and pro-caspase-1—cleaves pro-caspase-1 into its active form upon assembly. This activation subsequently promotes the processing and secretion of the proinflammatory cytokines IL-1β and IL-18 (6). Previous studies have established NLRP3 inflammasome activation as a critical mediator in the pathogenesis of various kidney diseases, including acute kidney injury (AKI) (7), lupus nephritis (8), and DKD (9). Our earlier work further demonstrated that NLRP3 inflammasome activation promotes TECs injury in chronic kidney disease (CKD) patients with hyperlipidemia (10). Nevertheless, the specific regulatory mechanisms governing NLRP3 activation in TECs under diabetic conditions remain incompletely elucidated.

Soluble epoxide hydrolase (sEH), an α/β-hydrolase fold enzyme, highly expressed in the liver, kidneys, and brain (11). This enzyme has been implicated in the pathogenesis of cardiovascular diseases, neurological disorders, and metabolic syndromes. Pharmacological inhibition of sEH has thus emerged as a promising therapeutic strategy for metabolic diseases (12). Epoxyeicosatrienoic acids (EETs), biologically active metabolites of arachidonic acid generated by cytochrome P450 (CYP) enzymes, exert multiple beneficial effects such as anti-hypertensive, anti-oxidative, and anti-inflammatory effects (13). However, endogenous EETs are rapidly hydrolyzed by sEH into less active dihydroxyeicosatrienoic acids (DHETs), and inhibiting sEH activity effectively stabilizes endogenous EET levels and enhances their beneficial effects (14). Previous studies have demonstrated that sEH inhibition and elevated levels of EETs attenuated inflammatory responses and tissue injury in models of viral myocarditis (15), adriamycin-induced nephropathy (16), acute lung injury (17). We have previously reported that sEH inhibition attenuates hyperglycemia-induced renal tubular injury through restoration of autophagic flux (18). However, it remains unclear whether inhibition of sEH exerts anti-inflammatory effects in hyperglycemia-induced renal injury — and in particular, whether it suppresses NLRP3 inflammasome activation via mitophagy in renal tubular cells during DKD.

In this study, we aimed to investigate the potential role of sEH in regulating renal tubular NLRP3 inflammasome activation in TECs during DKD and elucidated the underlying mechanisms, specifically focusing on sEH-mediated regulation of mitophagy in high glucose-stimulated HK-2 cells.

## 2 Materials and methods

### 2.1 Cell culture and treatment

HK-2 cells (human proximal tubular epithelial cells) were cultured as previously described (18). Cells were maintained in DMEM/F-12 medium (Gibco, USA) supplemented with 10% fetal bovine serum (PAN, Germany), 1000 U/L penicillin, and 100 μg/mL streptomycin at 37 °C in a 5% CO_2_ atmosphere. For glucose treatments, cells were exposed to low glucose (LG, 5.5 mM D-glucose), high glucose (HG, 30 mM D-glucose), or an osmotic control (5.5 mM glucose + 24.5 mM D-mannitol) for 24 h. In pharmacological experiments, HK-2 cells were treated under various conditions with the sEH inhibitor *trans*-4-[4-(3-adamantan-1-yl-ureido)-cyclohexyloxy]-benzoic acid (t-AUCB, 10 μM, ApexBio, USA), the NLRP3 inhibitor MCC950 (10 μM, MedChemExpress), the autophagy inducer rapamycin (Rapa, 10 μM, Selleck, S1039), the mitochondrial antioxidant MitoTEMPO (100 μM, MedChemExpress), or the autophagy inhibitor 3-methyladenine (3-MA, 5 nM, Selleck, S2767).

### 2.2 Animal experiments

C57BL/6 mice (6–8 weeks old) were supplied by the Animal Center of Chongqing Medical University (Chongqing, China) and maintained in a specific pathogen-free (SPF) facility, allowing free access to water and chow. After one week of acclimatization, mice were randomly divided into three groups: Control group (Ctr, fed with normal diet, n=8), diabetic kidney disease model group (DKD, fed with high-fat diet, n=8) and DKD+sEH inhibitor group (DKD+t-AUCB, fed with high-fat diet, n=8). The normal diet (#D12450J, 10 kcal% from fat) and high-fat diet (D12492, 60 kcal% from fat) were purchased from Research Diets. After 4 weeks, the mice fed a high-fat diet were intraperitoneally injected with 50 mg/kg streptozotocin (STZ, TargetMol T1507, USA) for 5 consecutive days, while control mice received vehicle injections (0.1 M citrate buffer, PH 4.5). One week after the STZ injection, the mice with fasting blood glucose levels ≥16.7 mmol/L were diagnosed with T2DM. At 16 weeks, the DKD + t-AUCB group received continuous t-AUCB administration via drinking water (2 mg/L) for 4 weeks. At the end of the experimental period, all mice were euthanized with intraperitoneal injection of pentobarbital sodium solution (100 mg/kg), blood and kidney tissue samples were collected for subsequent analysis. All animal procedures were approved by the Ethics Committee of Chongqing Medical University (IACUC-CQMU-2025-0253).

### 2.3 Western blotting

Cultured cells and kidney tissues were lysed with RIPA lysis buffer supplemented with protease inhibitors (Beyotime, China) and centrifuged at 12,000 rpm for 10 min at 4°C. Equal amounts of protein from each sample were loaded and separated by 8%–12% SDS-PAGE, electrophoretically transferred to PVDF membranes (Millipore, USA), blocking with 5% milk and incubated separately with the following primary antibodies: mouse anti-sEH (Santa Cruz, sc-166961), rabbit anti-Lamp2 (CST, #49067), rabbit anti-LC3 (CST, #4108), mouse anti-p62 (Santa Cruz, sc-48402), rabbit anti-NLRP3 (Abmart, #P60622R3), mouse anti-caspase1 (Santa Cruz, sc-56036), rabbit anti-IL-18 (Abcam, ab191860), rabbit anti-PINK1(Proteintech,23274-1-AP), rabbit anti-Parkin (Proteintech,14060-1-AP), and mouse anti-β-actin (Proteintech, 66009-1-Ig). The following day, the membranes were incubated with appropriate HRP-conjugated secondary antibodies for 1 h at room temperature, and protein bands were visualized using an enhanced chemiluminescence detection system (GE Healthcare, Piscataway, NJ, USA).

### 2.4 Immunofluorescence staining

HK-2 cells were seeded in 12-well plates and subjected to the indicated treatments. Cells were incubated with either MitoTracker Red (50 nM, M22425, Invitrogen, USA) or LysoTracker Red DND-99 (75 nM, Invitrogen, L7528) in serum-free medium for 30 min at 37 °C. After washing with phosphate-buffered saline (PBS), cells were fixed with 4% paraformaldehyde for 30 min, permeabilized with 0.2% Triton X-100 for 3 min, and blocked with 5% bovine serum albumin (BSA) for 1 h. The cells were then incubated overnight at 4 °C with the following primary antibodies diluted in blocking buffer: rabbit anti-Parkin (1:200), rabbit anti-NLRP3 (1:200), rabbit anti-LC3 (1:200), mouse anti-p62 (1:200), mouse anti-Lamp2 (1:200), and mouse anti-caspase-1 (1:200). After PBS washes, samples were incubated for 1 h at room temperature with corresponding secondary antibodies: Alexa Fluor 488-conjugated anti-rabbit IgG (1:400, Invitrogen), Alexa Fluor 488-conjugated anti-mouse IgG (1:400, Invitrogen), Alexa Fluor 594-conjugated anti-rabbit IgG (1:400, Invitrogen), or Alexa Fluor 594-conjugated anti-mouse IgG (1:400, Invitrogen). Nuclei were counterstained with DAPI, and fluorescent images were acquired using a fluorescence microscope.

### 2.5 Cell counting kit-8 assay

Cell viability was assessed using the CCK-8 colorimetric assay (Sigma-Aldrich). Briefly, HK-2 cells were seeded in 96-well plates and subjected to the indicated treatments. Following treatment, cells were incubated with CCK-8 reagent (100 μL per well) for 2 hours at 37 °C in a 5% CO_2_ atmosphere. Absorbance was then measured at 450 nm using a Varioskan Flash multimode microplate reader (Thermo Scientific).

### 2.6 Measurement of mitochondrial membrane potential

The mitochondrial membrane potential was assessed using the JC-1 fluorescent probe (Beyotime, China). HK-2 cells were seeded in 12-well plates and treated as indicated. After washing with PBS, cells were incubated with 10 μg/mL JC-1 for 20 min at 37 °C. For tissue analysis, fresh-frozen kidney sections (4 μm thickness) were stained following the same protocol. Following incubation, samples were washed with JC-1 staining buffer and immediately visualized under a fluorescence microscope. The ΔΨm was expressed as the ratio of red fluorescence (JC-1 aggregates) to green fluorescence (JC-1 monomers), with higher values indicating greater mitochondrial membrane potential.

### 2.7 Measurement of mitochondrial ROS

Mitochondrial ROS levels were detected using the MitoSOX™ Red fluorescent probe (Invitrogen, M36008). HK-2 cells seeded in 12-well plates were treated as indicated, then incubated with 5 μM MitoSOX™ Red and 50 nM MitoTracker Green (Beyotime, China) in the dark for 20 min at 37 °C. Fresh-frozen kidney sections (4 μm thickness) were similarly stained with 5 μM MitoSOX™ Red under identical conditions. After washing with PBS, fluorescence signals from both cells and tissue sections were immediately visualized using a fluorescence microscope.

### 2.8 GFP-LC3 lentivirus transfection

HK-2 cells were transduced with GFP-LC3 lentivirus (LV-MAP1LC3B, 3905-1, Genechem) at a suitable multiplicity of infection (MOI) of 20, following the manufacturer’s protocol. After 48 hours of infection, HK-2 cells were selected with 2 μg/mL puromycin for 5 days to eliminate non-transduced cells. The medium containing puromycin was replaced every 2 days. Stable expression of GFP-LC3 was confirmed by fluorescence microscopy and subsequently utilized for subsequent experiments.

### 2.9 Immunohistochemical staining

Renal tissue slices (4-μm thickness) were prepared from paraffin-embedded kidney samples. After deparaffinization and rehydration through a graded ethanol series, antigen retrieval was conducted by microwave heating in sodium citrate buffer for 15 minutes. Endogenous peroxidase activity was quenched with 0.3% H_2_O_2_ for 15 minutes, followed by blocking with normal goat serum for 15 minutes. Sections were then incubated overnight at 4 °C with the following primary antibodies: anti-sEH (1:200), anti-Lamp2 (1:200), anti-LC3 (1:200), anti-p62 (1:200), anti-NLRP3 (1:200), anti-caspase-1 (1:200), anti-IL-18 (1:200), anti-PINK1 (1:200), and anti-Parkin (1:200). After PBS washes, sections were incubated with biotinylated secondary antibody (Zhongshan Golden Bridge Inc., China) for 30 minutes, developed with 3,3′-diaminobenzidine (DAB), and counterstained with hematoxylin. Following dehydration through an ethanol series, images were captured using a light microscope. Protein expression was quantified using ImageJ software by measuring the stained area and integrated optical density (IOD). The average optical density (AOD), calculated as IOD/area, was used for statistical analysis.

### 2.10 Single-cell data

Using the Kidney Interactive Transcriptomics (KIT) database (https://www.humphreyslab.com/SingleCell/), we analyzed single-nucleus RNA sequencing data from the study by Wilson et al. (19) to assess *EPHX2* expression in kidney cells from patients with DKD (n=3) and healthy controls (n=3).

### 2.11 Enzyme-linked immunosorbent assay

The levels of IL-1β (EK0392, Boster Biological Technology, Wuhan, China) and IL-18 (EK0864, Boster Biological Technology, Wuhan, China) in the supernatants of HK-2 cells were quantified using commercial ELISA kits, according to the manufacturer’s instructions. Absorbance was measured at 450 nm using a microplate reader.

### 2.12 Measurement of sEH enzymatic activity

EETs can be hydrolyzed into DHETs by the soluble epoxide hydrolase (sEH) enzyme. The conversion of EETs to DHETs by sEH is regioselective, with 14,15-EET as the preferred substrate. Thus, the 14,15-EET/DHET ratio was used as an indicator of sEH activity (20). The levels of 14,15-EET and 14,15-DHET, as well as the 14,15-EET/14,15-DHET ratio in urine of mice and HK-2 cells, were quantified using a commercial ELISA kit (DH2, Detroit R&D) according to the manufacturer’s instructions.

### 2.13 Statistical analysis

Quantitative data are presented as mean ± SEM. Statistical analyzes were performed using GraphPad Prism software (version 8.0). Differences between two groups were assessed using *t*-test, while comparisons among multiple groups were analyzed by one-way analysis of variance (ANOVA) followed by Tukey’s *post hoc* test. Bivariate correlation analysis was performed using Pearson’s correlation analysis with Image J software. A p-value below 0.05 was considered statistically significant.

## 3 Result

### 3.1 Tubular sEH expression was significantly upregulated in kidney during DKD

**Figure 1:**
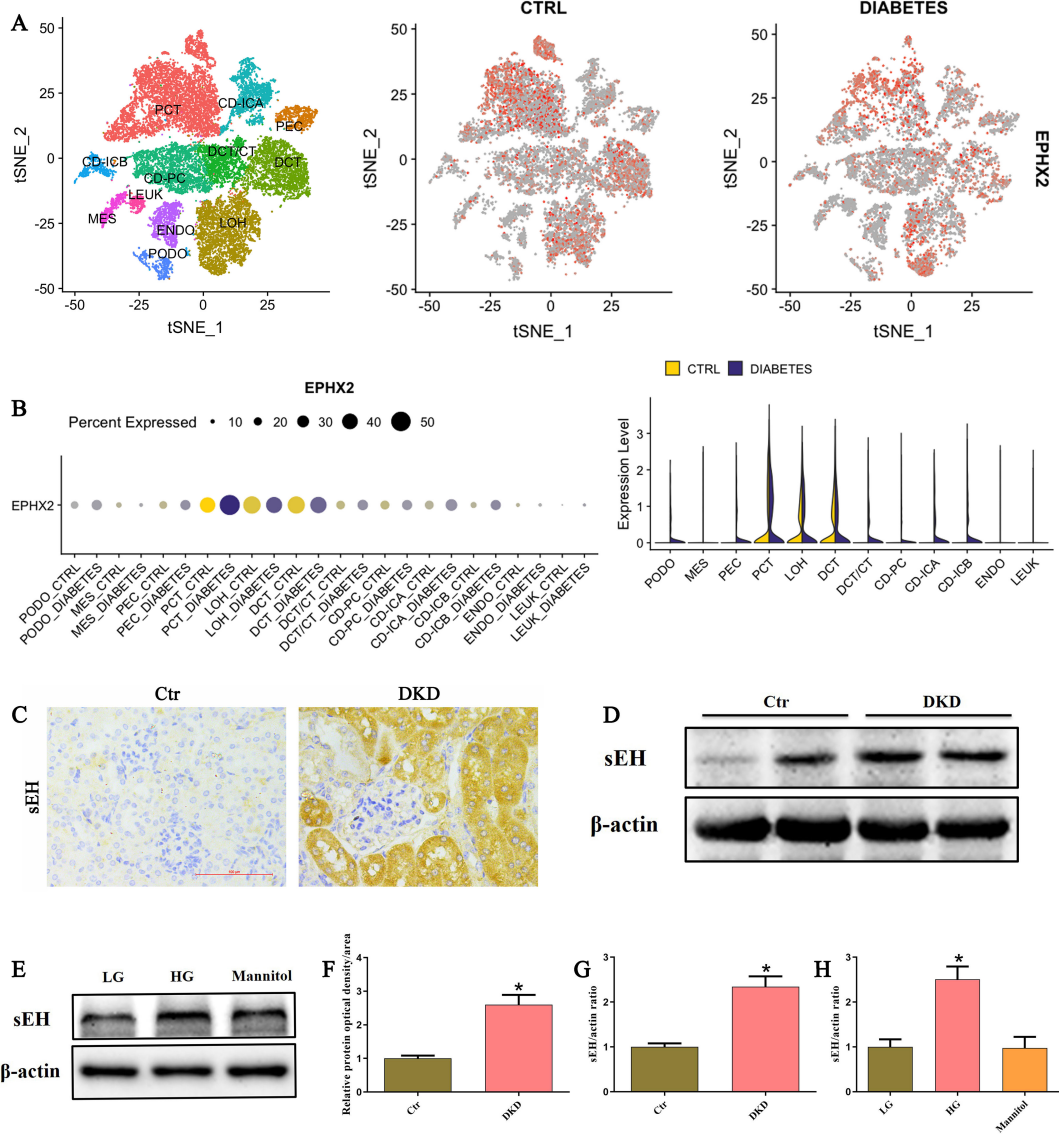
Tubular sEH expression was significantly upregulated in kidney during DKD. **(A, B)** scRNA-seq analysis revealed that *EPHX2* was mainly expressed in tubular epithelial cells. t-SNE plots showing human kidney cell clusters and the expression of *EPHX2* (https://www.humphreyslab.com/SingleCell/). PCT, proximal convoluted tubule; CFH, complement factor H; LOH, loop of Henle; DCT, distal convoluted tubule; CT, connecting tubule; CD, collecting duct; PC, principal cell; IC, intercalated cell; PODO, podocyte; ENDO, endothelium; MES, mesangial cell; LEUK, leukocyte. **(C)** Representative IHC images of sEH protein expression in the kidneys of control and DKD mice. **(D)** Western blot analysis of sEH protein expression in the kidneys of control and DKD mice. **(E)** Western blot analysis of sEH protein expression in HK-2 cells after treated with low- glucose, high- glucose, or mannitol for 24 h. **(F)** Quantification analysis of sEH protein expression from **(C)** (n = 6 mice per group, *P < 0.05 vs. Ctr). **(G)** Densitometric analysis of protein bands from **(D)** (n = 6 mice per group, *P < 0.05 vs. Ctr). **(H)** Densitometric analysis of protein bands from **(E)** (n = 3 independently repeated cultures, *P < 0.05 vs. LG).

To investigate the expression pattern of soluble epoxide hydrolase (sEH, encoded by *EPHX2*) in diabetic kidney disease, we first analyzed published single-cell RNA-sequencing datasets from the study by Wilson et al. (19). As shown in **Figures 1A, B**, *EPHX2* expression was predominantly elevated in tubular epithelial cells (TECs) of human DKD kidneys. We next validated these findings in a mice model of DKD. Immunohistochemical staining revealed increased sEH expression localized primarily to TECs (**Figures 1C, F**), which was further confirmed by western blot analysis of whole-kidney lysates (**Figures 1D, G**). To determine whether high glucose directly modulates sEH expression *in vitro*, we treated HK-2 cells with low glucose (LG, 5 mM), high glucose (HG, 30 mM), or an osmotic control (mannitol, 30 mM) for 24 hours. HG treatment significantly increased sEH protein levels compared to both LG and mannitol conditions (**Figures 1E, H**). Collectively, these results demonstrate that upregulated tubular sEH expression is a common feature both *in vivo* and vitro during DKD.

### 3.2 Inhibition of sEH alleviated renal tubular injury and improved kidney function in DKD mice

**Figure 2:**
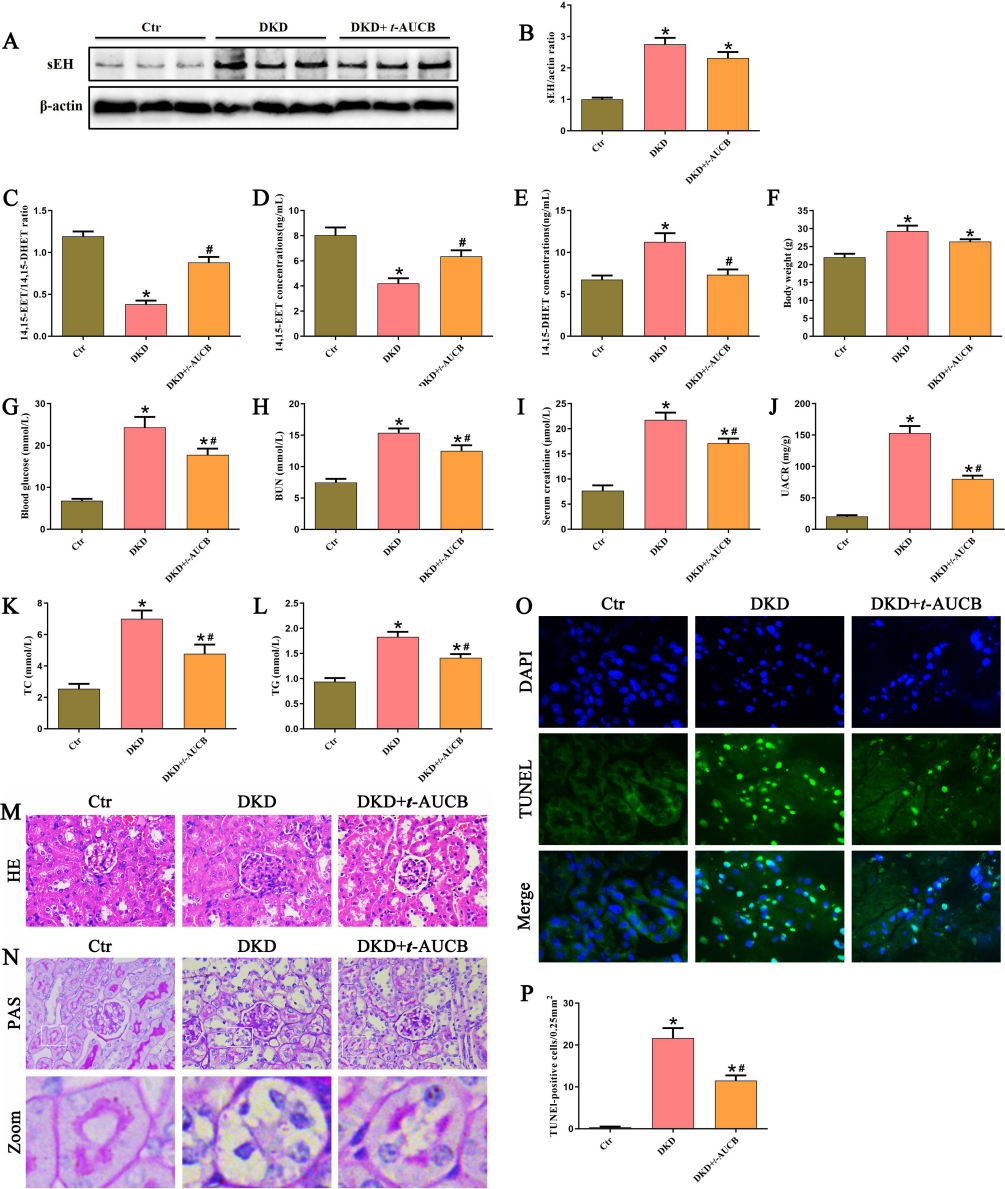
Inhibition of sEH alleviated renal tubular injury and improved kidney function in DKD mice. **(A)** Western blot analysis of sEH protein expression in the kidneys of different groups. **(B)** Densitometric analysis of protein bands from **(A)**. **(C-E)** ELISA kit analysis of the ratios of 14,15-EET/14,15-DHET, 14,15-EET and 14,15-DHET levels in different groups of mice. **(F-L)** The changes of Body weight, blood glucose, BUN, SCr, UACR, TC, and TG levels in different groups of mice. **(M)** Representative images of HE staining in the kidneys of different groups. **(N)** Representative images of PAS staining in the kidneys of different groups. **(O, P)** Representative images and quantification of TUNEL staining in the kidneys of different groups. (n = 6 mice per group, *P < 0.05 vs. Ctr, ^#^*P* < 0.05 vs. DKD).

To further investigate the role of sEH in diabetic kidney disease *in vivo*, we administered the sEH inhibitor *t*-AUCB to streptozotocin (STZ)-induced DKD mice. Immunoblot analysis confirmed significant upregulation of renal sEH expression in DKD mice (**Figure 2A**). While *t*-AUCB treatment did not significant affect sEH protein levels (**Figures 2A, B**), it effectively inhibited enzymatic activity, as demonstrated by an increased 14,15-EET/14,15-DHET ratio (**Figure 2C**). Consistent with this inhibition, *t*-AUCB administration significantly elevated urinary 14,15-EET levels (**Figure 2D**) and reduced 14,15-DHET levels (**Figure 2E**) compared to untreated DKD mice. Additionally, DKD mice developed characteristic metabolic disturbances, including elevated Body weight (**Figure 2F**), blood glucose (**Figure 2G**), blood urea nitrogen (BUN) (**Figure 2H**), serum creatinine (SCr) (**Figure 2I**), urine albumin-creatinine ratio (UACR) (**Figure 2J**), total cholesterol (TC) (**Figure 2K**), and triglyceride (TG) (**Figure 2L**) levels compared to control mice. *t*-AUCB treatment significantly ameliorated these parameters, though it did not significantly affect body weight. Histopathological analysis revealed that DKD mice developed substantial glomerular hypertrophy, mesangial expansion with matrix accumulation (**Figure 2M**), and marked tubular epithelial cell disruption (**Figure 2N**). These pathological changes were markedly attenuated by *t*-AUCB intervention. Furthermore, TUNEL staining showed a significant increase in apoptotic cells within tubular epithelial compartments of DKD mice, which was effectively reduced by *t*-AUCB treatment (**Figures 2O, P**). Collectively, these findings demonstrate that pharmacological inhibition of sEH with *t*-AUCB significantly alleviates renal tubular injury, improves renal functional parameters, and reduces pathological features in diabetic mice.

### 3.3 Inhibition of sEH attenuated HG-induced HK-2 cells injury

**Figure 3:**
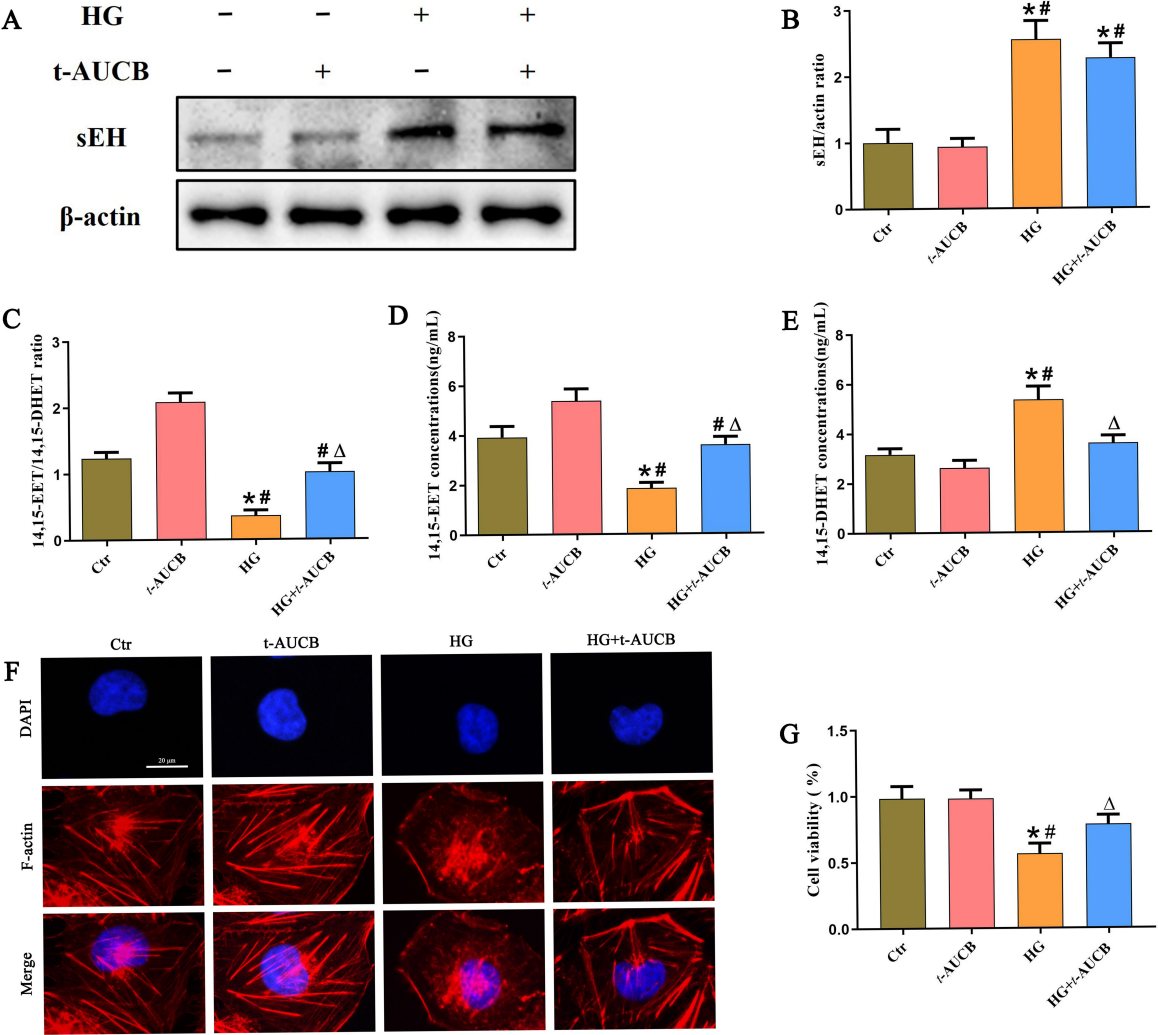
Inhibition of sEH attenuated HG-induced HK-2 cells injury. **(A)** Western blot analysis of sEH protein expression in HG stimulated HK-2 cells treated with t-AUCB for 24 h. **(B)** Densitometric analysis of protein bands from **(A)**. **(C-E)** ELISA kit analysis of the ratios of 14,15-EET/14,15-DHET, and the levels of 14,15-EET, 14,15-DHET in HK-2 cells. **(F)** Representative fluorescence-labeled phalloidin staining images showing the cytoskeletal changes in HK-2 cells incubated with or without t-AUCB under HG exposure. **(G)** CCK-8 assay of HG-induced HK-2 cells treated with or without *t*-AUCB under HG exposure. (n = 3 independently repeated cultures, *P < 0.05 vs. LG, ^#^*P* < 0.05 vs. *t*-AUCB, ^Δ^*P* < 0.05 vs. HG).

To investigate whether sEH inhibition protects against high glucose-induced renal tubular injury *in vitro*, we treated HK-2 cells with the sEH inhibitor *t*-AUCB during HG exposure. Notably, while t-AUCB did not alter sEH protein expression (**Figures 3A, B**), it effectively suppressed its enzymatic activity, as confirmed by a significant increase in the 14,15-EET/14,15-DHET ratio (**Figure 3C**), elevated 14,15-EET levels (**Figure 3D**), and reduced 14,15-DHET levels (**Figure 3E**) in HG-stimulated HK-2 cells. Phalloidin staining revealed that HG exposure induced severe cytoskeletal disruption, characterized by disorganized and reduced actin stress fibers compared to control cells (**Figure 3F**). *t*-AUCB treatment markedly preserved actin cytoskeleton integrity under these conditions. Consistent with these morphological improvements, the CCK-8 assay demonstrated that *t*-AUCB significantly enhanced the viability of HG-treated HK-2 cells (**Figure 3G**). These results indicate that pharmacological inhibition of sEH alleviates high glucose-induced cell injury and promotes cell survival in renal tubular epithelial cells.

### 3.4 sEH inhibition suppressed NLRP3 inflammasome activation in HG-treated HK-2 cells

**Figure 4:**
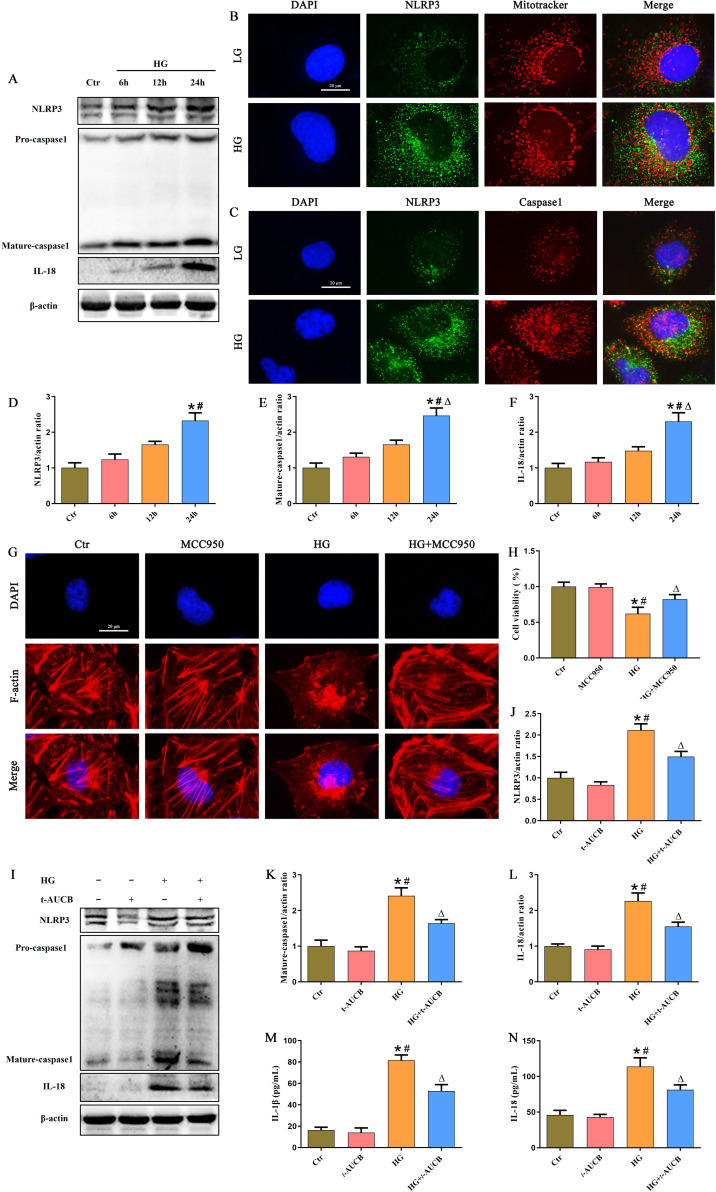
Inhibition of sEH suppressed the activation of NLRP3 inflammasome in HG -treated HK-2 cells. **(A)** Western blot analysis of NLRP3, Caspase-1, and IL-18 protein expression in HK-2 cells after treated with HG for different durations. **(B)** Representative immunofluorescence double-staining images showing the colocalization of NLRP3 (green) with Mito tracker (red) in HK-2 cells under HG exposure. **(C)** Representative immunofluorescence double-staining images showing the colocalization of NLRP3 (green) with Caspase1 (red) in HK-2 cells under HG exposure. **(D-F)** Densitometric analysis of protein bands from **(A)**. **(G)** Representative fluorescence-labeled phalloidin staining images showing the cytoskeletal changes in HK-2 cells incubated with or without MCC950 under HG exposure. **(H)** CCK-8 assay of HG-induced HK-2 cells treated with or without MCC950 under HG exposure. **(I)** Western blot analysis of NLRP3, Caspase-1, and IL-18 protein expression in HK-2 cells treated with or without t-AUCB under HG exposure. **(J-L)** Densitometric analysis of protein bands from **(I)**. **(M-N)** The levels of IL-1β and IL-18 in the conditioned media from HK-2 cells were quantified by ELISA. (n = 3 independently repeated cultures, *P < 0.05 vs. LG, ^#^*P* < 0.05 vs. *t*-AUCB, ^Δ^*P* < 0.05 vs. HG).

The NLRP3 inflammasome has been increasingly implicated in the pathogenesis of DKD (21). To determine the effect of HG on the NLRP3 inflammasome in HK-2 cells, we treated HK-2 cells with HG at different times and found that the expression of NLRP3, Mature caspase-1 and IL-18 in HK-2 cells increased in a time-dependent manner, as evidenced by western blot analysis (**Figures 4A, D–F**). Given the established role of mitochondria as platforms for NLRP3 inflammasome assembly, we next investigated the subcellular localization of NLRP3 under HG stimulation. In control cells, NLRP3 was diffusely distributed in the cytoplasm with minimal mitochondrial colocalization. However, HG treatment promoted significant mitochondrial translocation of NLRP3, as demonstrated by increased overlap with the mitochondrial marker (**Figure 4B**). Furthermore, we observed enhanced colocalization between NLRP3 and Caspase-1 following HG exposure (**Figure 4C**), indicating that HG initiated the assembly and formation of NLRP3 inflammasome complexes in HK-2 cells. Furthermore, to examine the effect of NLRP3 inflammasome activation on the HK-2 cells, the cells were treated with MCC950, a NLRP3 antagonist, for 24 h, and we found MCC950 treatment significantly attenuated cytoskeletal disruption (**Figure 4G**) and restored cell viability (**Figure 4H**) in HG-induced HK-2 cells, indicating that NLRP3 inflammasome activation mediated HG-induced HK-2 cells injury. Finally, to determine whether sEH inhibition affects NLRP3 inflammasome signaling, we assessed the expression levels of NLRP3, Mature-caspase1 and IL-18 proteins. Immunoblot analysis demonstrated that *t*-AUCB substantially suppressed the HG-induced upregulation of NLRP3, Mature-caspase-1, and IL-18 (**Figures 4I–L**). ELISA confirmed that *t*-AUCB decreased IL-1β and IL-18 secretion by HK-2 cells under HG conditions (**Figures 4M, N**). Collectively, these findings indicated that sEH inhibition attenuated HG-induced HK-2 cells injury by suppressing NLRP3 inflammasome activation.

### 3.5 Inhibition of sEH restored autophagy flux and enhanced mitophagy in HG-stimulated HK-2 cells

**Figure 5:**
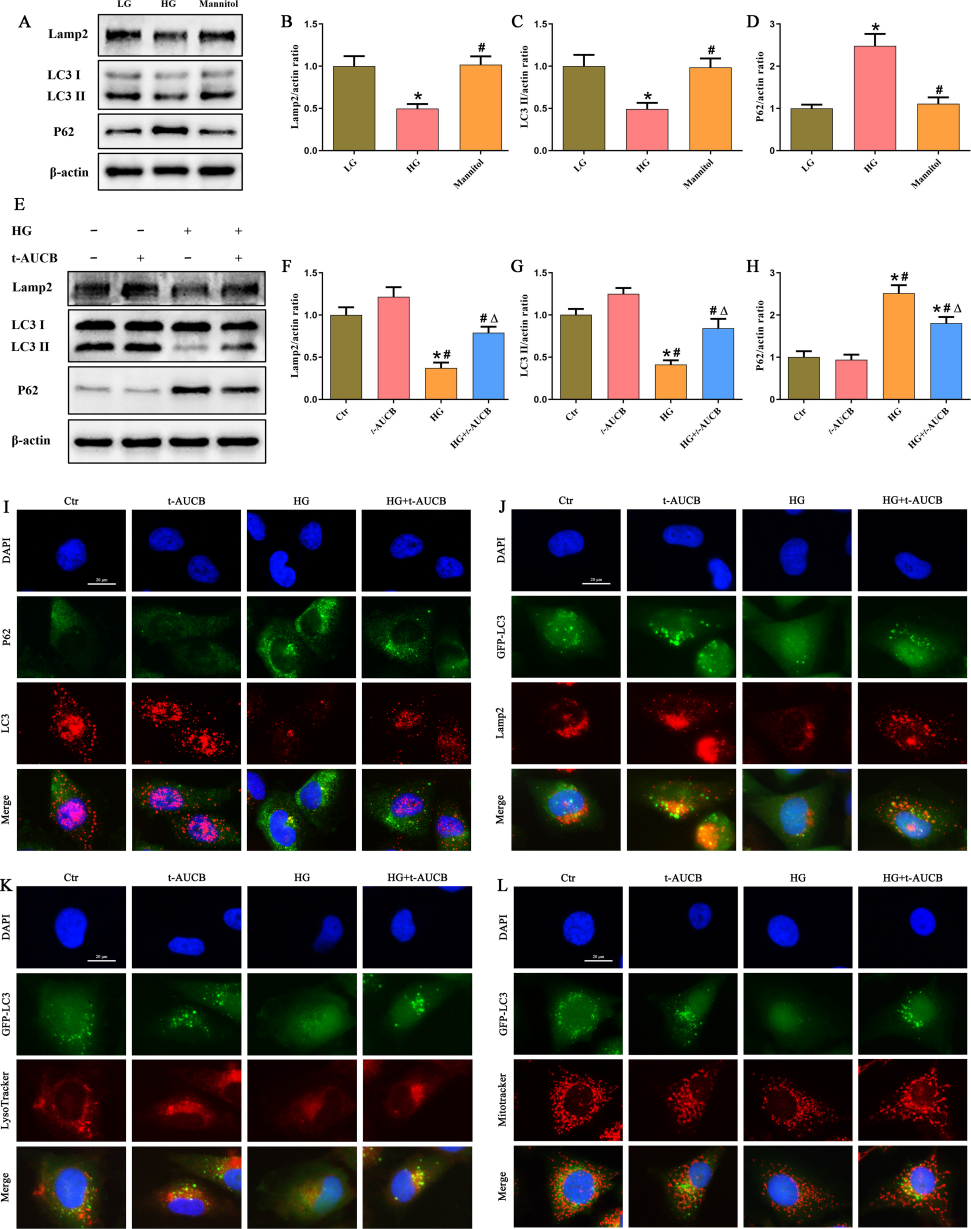
Inhibition of sEH improved autophagy flux and enhanced mitophagy in HG-induced HK-2 cells. **(A)** Western blot analysis of Lamp2, LC3 and P62 protein expression in HK-2 cells after treated with low- glucose, high- glucose, or mannitol for 24 h. **(B-D)** Densitometric analysis of protein bands from **(A)**. **(E)** Western blot analysis of Lamp2, LC3 and P62 protein expression in HK-2 cells treated with or without t-AUCB under HG exposure. **(F-H)** Densitometric analysis of protein bands from **(E)**. **(I)** Representative immunofluorescence double-staining images showing the colocalization of P62 (green) with LC3 (red) in HK-2 cells treated with or without *t*-AUCB under HG exposure. **(J)** Representative immunofluorescence double-staining images showing the colocalization of GFP-LC3 (green) with Lamp2 (red) in HK-2 cells treated with or without *t*-AUCB under HG exposure. **(K)** Representative immunofluorescence double-staining images showing the colocalization of GFP-LC3 (green) with Lysotracker (red) in HK-2 cells treated with or without *t*-AUCB under HG exposure. **(L)** Representative immunofluorescence double-staining images showing the colocalization of GFP-LC3 (green) with Mitotracker (red) in HK-2 cells treated with or without *t*-AUCB under HG exposure. (n = 3 independently repeated cultures, *P < 0.05 vs. LG, ^#^*P* < 0.05 vs. *t*-AUCB, ^Δ^*P* < 0.05 vs. HG).

We next examined whether sEH inhibition modulates autophagy flux in renal tubular cells under HG conditions. Western blot analysis revealed that high glucose exposure significantly reduced the expression of lysosomal-associated membrane protein 2 (Lamp2) and lipidated LC3 (LC3-II), while increasing levels of the autophagic substrate p62 (**Figures 5A–D**). These alterations were substantially reversed by *t*-AUCB treatment (**Figures 5E–H**), suggesting improved autophagic flux. Consistent with these findings, immunofluorescence staining demonstrated that *t*-AUCB increased the formation of LC3-positive puncta and reduced p62 accumulation in HG-stimulated HK-2 cells (**Figures 5I**). Furthermore, *t*-AUCB enhanced the colocalization of GFP-LC3 with Lamp2 (**Figure 5J**) or LysoTracker Red (**Figure 5K**), indicating that *t*-AUCB promoted autophagosome-lysosome fusion and autolysosome formation. To specifically assess mitophagy, we examined the colocalization of GFP-LC3 with MitoTracker Red. *t*-AUCB treatment significantly increased the overlap between these markers (**Figure 5L**), demonstrating enhanced formation of mitophagosomes in HG-exposed HK-2 cells. The PINK1/Parkin-dependent pathway is the most recognized mechanisms for initiating mitophagy. We further found that t-AUCB treatment significantly upregulated the mitophagy-related protein levels of PINK1 and Parkin (**Supplementary Figure 1A**), and increased the colocalization of Parkin and MitoTracker Red (**Supplementary Figure 1D, E**) in HG-exposed HK-2 cells. These results collectively indicated that sEH inhibition restored autophagic flux and promoted PINK1/Parkin-mediated mitophagy under high glucose conditions.

### 3.6 Activation of Mitophagy mitigated HG‐induced mitochondrial damage and NLRP3 inflammasome activation in HK-2 cells

**Figure 6:**
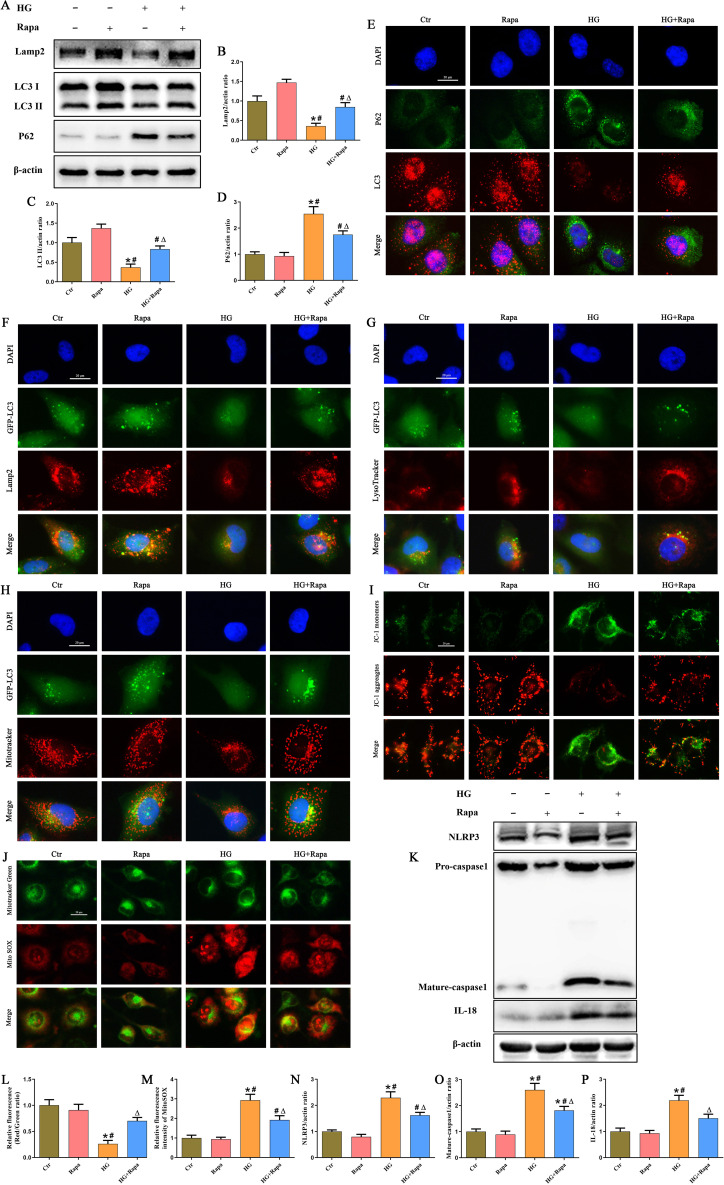
Activation of Mitophagy mitigated HG‐induced mitochondrial damage and NLRP3 inflammasome activation in HK-2 cells. **(A)** Western blot analysis of Lamp2, LC3 and P62 protein expression in HK-2 cells treated with or without Rapa under HG exposure. **(B-D)** Densitometric analysis of protein bands from **(A)**. **(E)** Representative immunofluorescence double-staining images showing the colocalization of P62 (green) with LC3 (red) in HK-2 cells treated with or without Rapa under HG exposure. **(F)** Representative immunofluorescence double-staining images showing the colocalization of GFP-LC3 (green) with Lamp2 (red) in HK-2 cells treated with or without Rapa under HG exposure. **(G)** Representative immunofluorescence double-staining images showing the colocalization of GFP-LC3 (green) with Lysotracker (red) in HK-2 cells treated with or without Rapa under HG exposure. **(H)** Representative immunofluorescence double-staining images showing the colocalization of GFP-LC3 (green) with Mitotracker (red) in HK-2 cells treated with or without Rapa under HG exposure. **(I)** Representative fluorescence images of the mitochondrial membrane potential by JC-1 staining in HK-2 cells treated with or without Rapa under HG exposure. **(J)** Representative fluorescence images of MitoTracker Green and MitoSOX staining in HK-2 cells treated with or without Rapa under HG exposure. **(K)** Western blot analysis of NLRP3, caspase-1, and IL-18 protein expression in HK-2 cells treated with or without Rapa under HG exposure. **(L)** Quantification of the fluorescence intensities of JC-1 from **(I)**. **(M)** Quantification of the fluorescence intensities of MitoSOX from **(J)**. **(N-P)** Densitometric analysis of protein bands from **(K)**. (n = 3 independently repeated cultures, *P < 0.05 vs. LG, *^#^P* < 0.05 vs. *t*-AUCB, ^Δ^*P* < 0.05 vs. HG).

To investigate the functional relationship between autophagy and NLRP3 inflammasome regulation, we treated HK-2 cells with rapamycin (Rapa), a specific mTOR-dependent autophagy inducer. Immunoblot analysis demonstrated that Rapa significantly increased Lamp2 and LC3-II expression while reducing p62 accumulation in HG-stimulated cells (**Figures 6A–D**). Consistent with these findings, immunofluorescence revealed that Rapa enhanced LC3 puncta formation and decreased p62 fluorescence intensity in HG-induced HK-2 cells (**Figure 6E**). Furthermore, Rapa promoted the colocalization of GFP-LC3 with Lamp2 (**Figure 6F**) or LysoTracker Red (**Figure 6G**), indicating that Rapa enhanced autophagosome-lysosome fusion. These results confirm that Rapa restored autophagic flux in cells under high glucose conditions.

Accumulating evidence has demonstrated that mitochondrial injury plays an important role in the activation of the NLRP3 inflammasome, especially the ROS released by damaged mitochondria can activate the NLRP3 inflammasome (22). Mitophagy serves as a mechanism of mitochondrial quality control by removing damaged mitochondria, thereby reducing ROS generation and inhibiting the activation of NLRP3 inflammasome (23). Our immunofluorescence staining showed that Rapa promoted mitophagy (**Figure 6H**), preserved mitochondrial membrane potential (ΔΨm) (**Figures 6I, L**), and reduced mitochondrial ROS (mtROS) production (**Figures 6J, M**) in HG-induced HK-2 cells. Importantly, Rapa treatment also suppressed NLRP3 inflammasome activation, as evidenced by reduced protein levels of NLRP3, Mature-caspase1 and IL-18 in HG-treated HK-2 cells (**Figures 6K, N–P**). Together, these data indicated that enhancing mitophagy with Rapa alleviated HG-induced mitochondrial damage, reduced mtROS production, and subsequently inhibited NLRP3 inflammasome activation in HK-2 cells under HG condition.

### 3.7 Inhibition of sEH promoted mitophagy and subsequently suppressed NLRP3 inflammasome activation through reducing mtROS production

**Figure 7:**
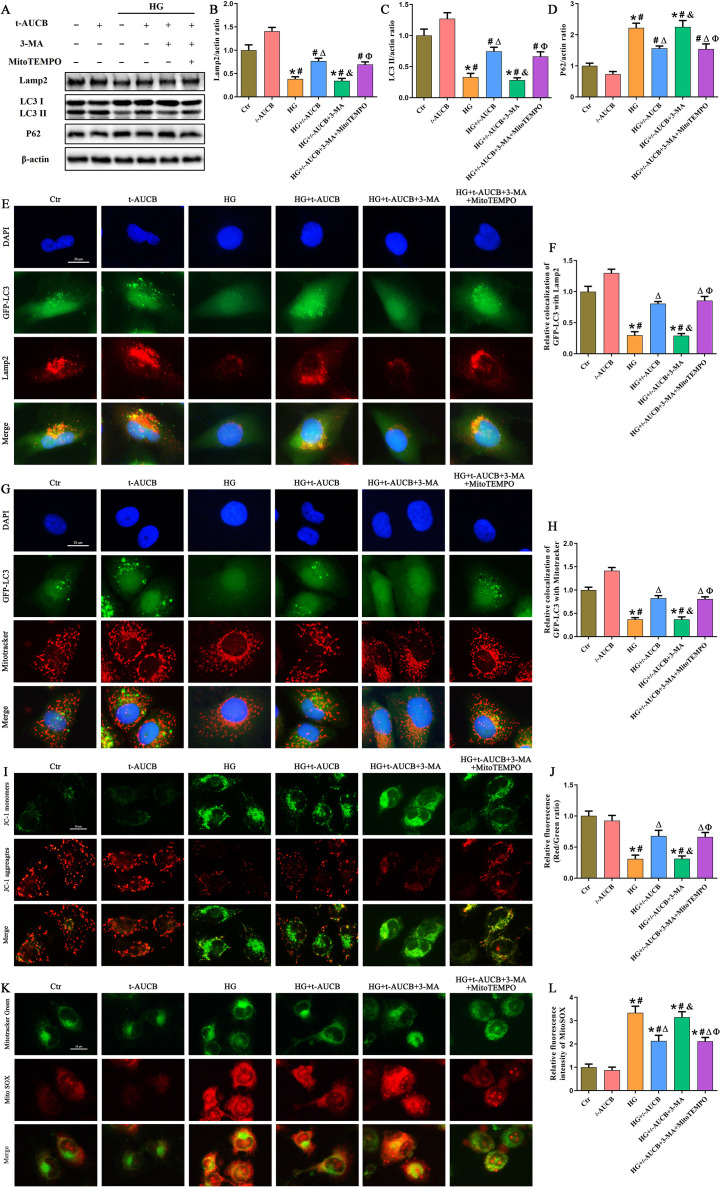
Inhibition of sEH promoted mitophagy and reduced mtROS production. **(A)** Western blot analysis of Lamp2, LC3 and P62 protein expression in HK-2 cells of different groups. **(B-D)** Densitometric analysis of protein bands from **(A)**. **(E)** Representative immunofluorescence double-staining images showing the colocalization of GFP-LC3 (green) with Lamp2 (red) in HK-2 cells of different groups. **(F)** Quantification of the colocalization of GFP-LC3 and Lamp2 in Figure **(E) (G)** Representative immunofluorescence double-staining images showing the colocalization of GFP-LC3 (green) with Mitotracker (red) in HK-2 cells of different groups. **(H)** Quantification of the colocalization of GFP-LC3 and Mitotracker in Figure **(G) (I)** Representative fluorescence images of the mitochondrial membrane potential by JC-1 staining in HK-2 cells of different groups. **(J)** Quantification of the fluorescence intensities of JC-1 from **(I)**. **(K)** Representative fluorescence images of MitoTracker Green and MitoSOX staining in HK-2 cells of different groups. **(L)** Quantification of the fluorescence intensities of MitoSOX from **(K)**. (n = 3 independently repeated cultures, *P < 0.05 vs. LG, ^#^*P* < 0.05 vs. *t*-AUCB, ^Δ^*P* < 0.05 vs. HG, ^&^*P* < 0.05 vs. HG+*t*-AUCB, ^Ф^*P* < 0.05 vs. HG+*t*-AUCB+3-MA).

To further clarify the specific mechanism by which sEH inhibition regulates NLRP3 inflammasome activation, HG-stimulated HK-2 cells treated with *t*-AUCB were further incubated with 3-MA, a class III phosphatidylinositol 3-kinase (PI3K)-blocking autophagy inhibitor. As shown in **Figures 7A–D**, 3-MA treatment significantly decreased the expression of Lamp2, LC3-II and increased the p62 protein expression in HG-treated HK-2 cells, indicating effective suppression of autophagic flux. Consistent with this, 3-MA attenuated *t*-AUCB-induced enhancement of autolysosome formation and mitophagy, as demonstrated by reduced colocalization of GFP-LC3 with Lamp2 (**Figures 7E, F**) and MitoTracker Red (**Figures 7G, H**), respectively. Furthermore, 3-MA abrogated the protective effects of *t*-AUCB on mitochondrial membrane potential (ΔΨm) (**Figures 7I, J**) and mtROS production (**Figures 7K, L**) in HG-treated HK-2 cells.

**Figure 8:**
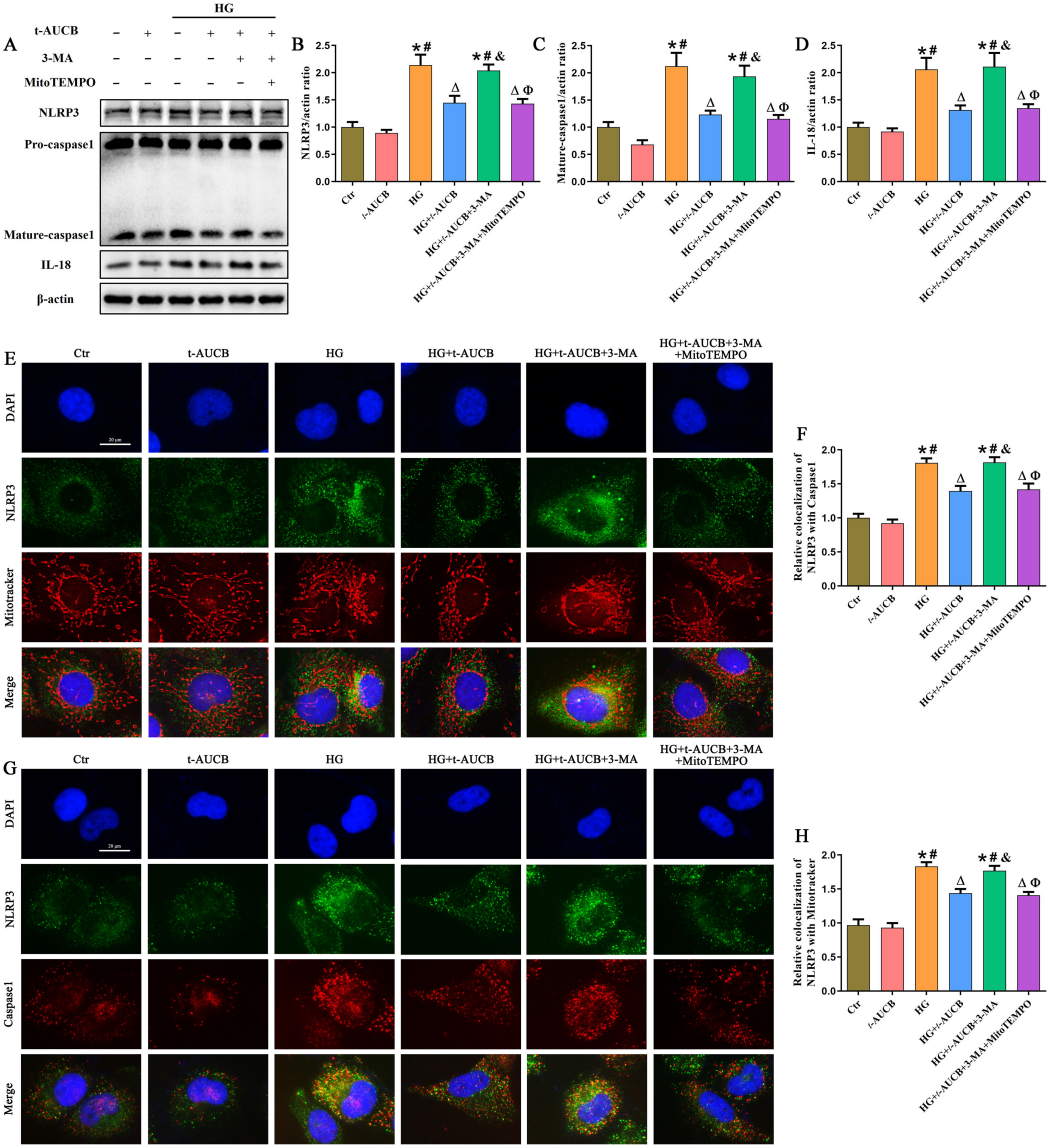
Inhibition of sEH suppressed HG-induced NLRP3 inflammasome activation through mitophagy-mediated reduction of mtROS production. **(A)** Western blot analysis of NLRP3, Caspase-1, and IL-18 protein expression in HK-2 cells given different treatments. **(B-D)** Densitometric analysis of protein bands from **(A)**. **(E)** Representative immunofluorescence double-staining images showing the colocalization of NLRP3 (green) with Mito tracker (red) in HK-2 cells given different treatments. **(F)** Quantification of the colocalization of NLRP3 and Mitotracker in Figure **(E)**. **(G)** Representative immunofluorescence double-staining images showing the colocalization of NLRP3 (green) with Caspase1 (red) in HK-2 cells given different treatments. **(H)** Quantification of the colocalization of NLRP3 and Caspase1 in Figure **(G)** (n = 3 independently repeated cultures, *P < 0.05 vs. LG, ^#^*P* < 0.05 vs. *t*-AUCB, ^Δ^*P* < 0.05 vs. HG, ^&^*P* < 0.05 vs. HG+*t*-AUCB, ^Ф^*P* < 0.05 vs. HG+*t*-AUCB+3-MA).

We next examined the effects of 3-MA on the anti-NLRP3 inflammasome efficacy of *t*-AUCB. Our results indicated that 3-MA reversed the inhibitory effects of *t*-AUCB on NLRP3 inflammasome assembly and activation in HG-treated HK-2 cells, as demonstrated by increased expression of NLRP3, Mature-caspase1 and IL-18 (**Figure 8A**), enhanced colocalization of NLRP3 with mitochondria or Caspase1 (**Figures 8E–G**). These findings demonstrated that sEH inhibition mitigated HG-triggered NLRP3 inflammasome activation via upregulation of mitophagy in HK-2 cells.

MtROS released from dysfunctional mitochondria is a direct activator of NLRP3 inflammatory vesicles (24). To further clarify the detail mechanism of sEH inhibiting NLRP3 inflammasome activation through mitophagy, 3-MA-treated HK-2 cells were further incubated with MitoTEMPO, a mitochondrial-targeted antioxidant. Interestingly, MitoTEMPO significantly reversed above changes, as evidenced by improved ΔΨm (**Figure 7I**), alleviated mtROS production (**Figure 7K**) and attenuated NLRP3 inflammasome assembly and activation (**Figures 8A–H**). Collectively, these results indicated that sEH inhibition suppressed HG-induced NLRP3 inflammasome activation through mitophagy-mediated reduction of mtROS production.

### 3.8 Inhibition of sEH promoted mitophagy and suppressed NLRP3 inflammasome activation in the kidneys of DKD mice

**Figure 9:**
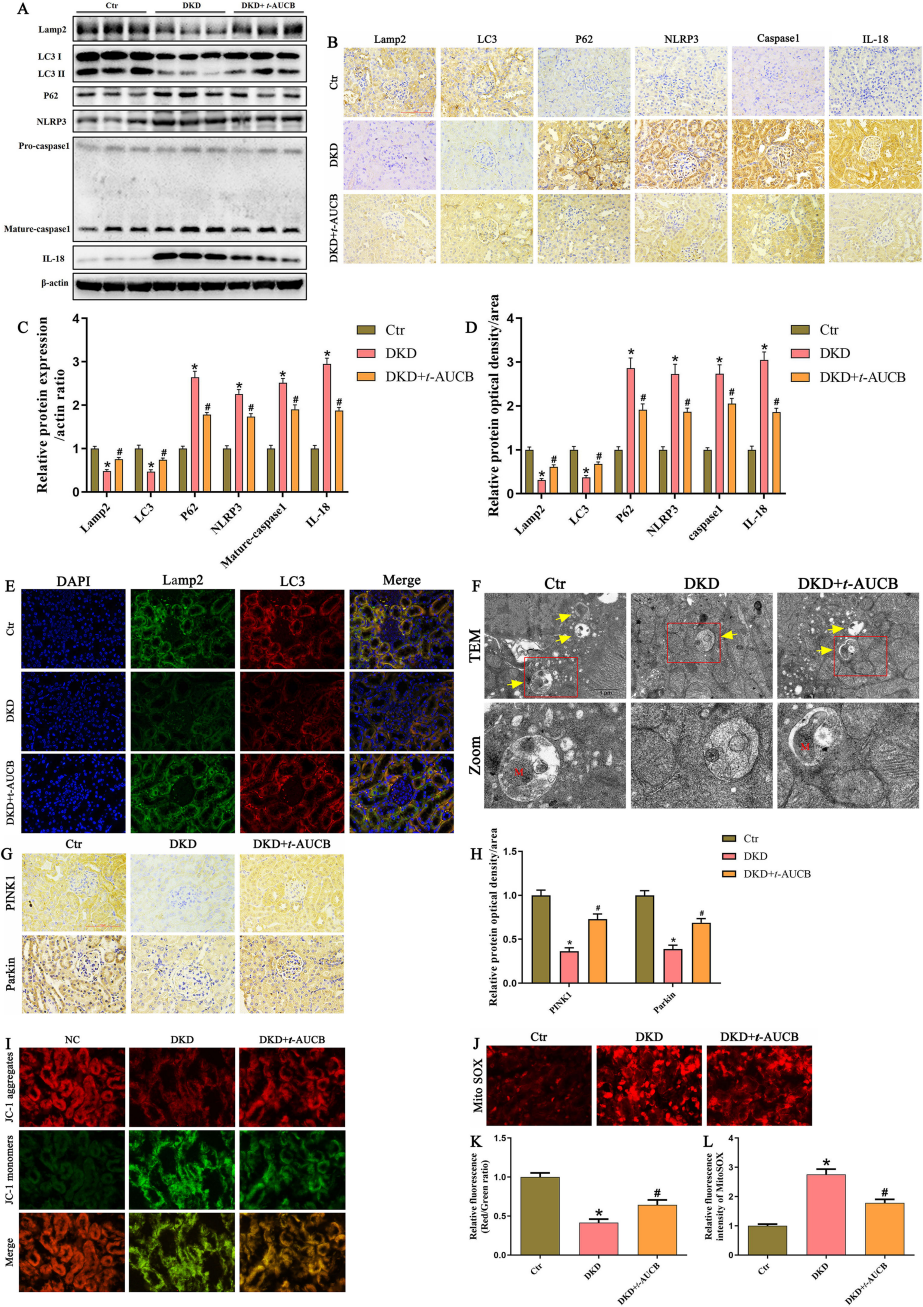
Inhibition of sEH promoted mitophagy and ameliorated NLRP3 inflammasome activation in the kidneys of T2DM mice. **(A)** Western blot analysis of Lamp2, LC3, P62, NLRP3, Caspase-1, and IL-18 protein expression in the kidneys of different groups. **(B)** Representative IHC images of Lamp2, LC3, P62, NLRP3, Caspase-1, and IL-18 protein expression in the kidneys of different groups. **(C)** Densitometric analysis of protein bands from **(A)**. **(D)** Quantification analysis of Lamp2, LC3, P62, NLRP3, Caspase-1, and IL-18 protein expression from **(B)**. **(E)** Representative immunofluorescence double-staining images showing the colocalization of Lamp2 (green) with LC3 (red) in the kidneys of different groups. **(F)** Representative TEM images of renal tubular epithelial cells in each group. Autophagosomes or autolysosomes were indicated by yellow arrows. M: Mitochondria. **(G)** Representative IHC images of PINK1 and Parkin protein expression in the kidneys of different groups. **(H)** Quantification analysis of PINK1 and Parkin protein expression from **(G)**. **(I)** Representative fluorescence images of the mitochondrial membrane potential by JC-1 staining in the kidneys of different groups. **(J)** Representative fluorescence images of MitoSOX staining in the kidneys of different groups. **(K)** Quantification of the fluorescence intensities of JC-1 from **(I)**. **(L)** Quantification of the fluorescence intensities of MitoSOX from **(J)**. (n = 6 mice per group, *P < 0.05 vs. Ctr, ^#^*P* < 0.05 vs. DKD).

Finally, we examined whether the renoprotective effects of sEH inhibition involved mitophagy and NLRP3 inflammasome regulation in DKD mice. Immunoblot analysis demonstrated that *t*-AUCB treatment increased the expression of Lamp2, LC3 II while reducing p62 expression, indicating *t*-AUCB restored autophagic activity in the kidneys of DKD mice (**Figure 9A**). Furthermore, our result revealed markedly elevated expression levels of NLRP3, Mature-caspase1, and IL-18 proteins in the kidneys of DKD mice. Notably, *t*-AUCB treatment effectively reduced the expression of these inflammatory markers (**Figures 9A, C**). Subsequently, immunohistochemical staining (**Figures 9B, D**) also confirmed the above-mentioned findings, indicating that inhibition of sEH upregulated autophagy level and suppressed the activation of the NLRP3 inflammasome in the kidneys of DKD mice.

Next, our immunofluorescence staining further revealed that *t*-AUCB treatment increased the colocalization of LC3 with Lamp2 (**Figure 9E**), suggesting that *t*-AUCB enhanced the autophagosome-lysosome fusion in the kidneys of DKD mice. Transmission electron microscopy (TEM) analysis revealed that *t*-AUCB treatment increased the formation of autophagosomes or autolysosomes in renal tubular epithelial cells of DKD mice; importantly, we observed the presence of partially degraded mitochondria within these autolysosomes (**Figure 9F**). Concurrently, *t*-AUCB administration upregulated the expression levels of PINK1 and Parkin (**Figures 9G, H**), and increased the colocalization of LC3 with VDAC as well as Parkin with VDAC (**Supplementary Figure 2A, B**), suggesting improved PINK1/Parkin mediated-mitophagy in the kidneys of DKD mice. Furthermore, immunofluorescence analysis showed that DKD mice treated with *t*-AUCB significantly restored the loss of ΔΨm (**Figures 9I, K**) and markedly alleviated mitochondrial ROS generation (**Figures 9J, L**) in the kidneys of DKD mice. Collectively, these results demonstrated that inhibition of sEH suppressed NLRP3 inflammasome activation by promoting mitophagy and reducing mtROS generation in the kidneys of DKD mice.

## 4 Discussion

In this study, we identified a novel renoprotective role for sEH inhibition in DKD. By analyzing published single-cell datasets from human DKD kidneys, we found that *EPHX2* is predominantly elevated in TECs, a finding corroborated by the upregulation of sEH in both high glucose (HG)-stimulated HK-2 cells and the kidneys of diabetic mice. Pharmacological inhibition of sEH attenuated mitochondrial dysfunction, reduced mtROS production, suppressed NLRP3 inflammasome activation, and ameliorated renal tubular injury under diabetic conditions both *in vivo* and *in vitro*. Mechanistically, we identified that sEH inhibition enhanced mitophagy, thereby improving mitochondrial function and decreasing mtROS-dependent NLRP3 inflammasome activation in DKD. Collectively, our findings demonstrate for the first time that inhibition of sEH exerts a protective role in DKD by promoting mitophagy and suppressing mtROS-mediated NLRP3 inflammasome activation. These results highlight sEH inhibition represents an attractive therapeutic strategy for DKD.

Inflammation plays a pivotal role in the initiation and progression of diabetic kidney disease (DKD), making it a promising therapeutic target for DKD treatment (5). The NLRP3 inflammasome is cytosolic multiprotein complex that activates caspase-1, thereby promoting the maturation and release of interleukin IL-1β and IL-18. This process plays a crucial role in innate immune defense and the maintenance of homeostasis. However, dysregulated activation of the NLRP3 inflammasome has been implicated in the pathogenesis of various inflammatory diseases, including diabetes (25), sepsis (26), Alzheimer’s disease (27), and atherosclerosis (28). Growing evidence specifically links tubular NLRP3 inflammasome activation to DKD development and progression. Activation of the NLRP3 inflammasome induces TECs injury and pyroptosis (29). Moreover, the subsequent secretion of pro-inflammatory cytokines, including IL-1β and IL-18, recruits circulating monocytes, macrophages, and lymphocytes into the renal tissue, thereby promoting renal inflammatory infiltration. Concurrently, macrophages preferentially polarize toward the pro-inflammatory M1 phenotype, which secretes cytokines such as TNF-α, IL-1β, and IL-6, further amplifying the local inflammatory response and exacerbating tissue injury in DKD (30). Therefore, inhibiting tubular NLRP3 inflammasome activation is an effective strategy for blocking DKD progression.

NLRP3 inflammasome activation is triggered by diverse stimuli, including K+ efflux, ATP, mitochondrial dysfunction, reactive oxygen species (ROS) and lysosomal damage, with mitochondria dysfunction being particularly implicated in this process (31). Emerging evidence indicates that mitochondria can function as a platform for NLRP3 inflammasome assembly, with mitochondrial antiviral-signaling protein (MAVS) (32) and mitofusin 2 (33) functioning as adaptor molecules that recruit NLRP3 to mitochondrial membranes. Importantly, mtROS released from damage mitochondria is a pivotal stimulus signal for NLRP3 inflammasome activation (24). Previous study has demonstrated that mtROS-TXNIP-NLRP3 biological axis activation is a critical contributor to renal tubular injury in DKD (34). Herein, we observed that HG exposure induced mitochondrial damage, mtROS generation, and NLRP3 inflammasome activation in HK-2 cells and diabetic mouse kidneys. Additionally, HG promoted NLRP3 translocation to mitochondria in HK-2 cells. These observations suggest that therapeutic strategies preserving mitochondrial integrity and reducing mtROS production represent promising approaches for suppressing NLRP3 inflammasome activation and ameliorating DKD.

sEH is a cytosolic enzyme, which primarily exerts its biological functions by regulating the bioavailability of EETs. It has recently garnered attention for its role in metabolic and inflammatory diseases (35–38). While sEH inhibition has been shown to suppress NLRP3 inflammasome activation in LPS-induced acute lung injury (39) and in high-fat diet-induced NAFLD (40). Its specific role in DKD, particularly in the context of mitochondrial quality control, remains elusive. In our study, we found that inhibition of sEH significantly attenuated mitochondrial dysfunction, suppressed NLRP3 inflammasome activation and alleviated renal TEC injury in both diabetic kidneys and HG-stimulated HK-2 cells.

Autophagy is an essential cellular catabolic process responsible for the degradation and recycling of cellular components, and mitophagy represents a selective form of autophagy that specifically eliminates damaged or dysfunctional mitochondria through lysosomal degradation (41). Mitophagy, a key mitochondrial quality control mechanism, is primarily regulated by the PINK1/Parkin pathway. Upon mitochondrial injury, PINK1 stabilizes on the outer mitochondrial membrane and recruits Parkin, which ubiquitinates substrates such as VDAC to tag damaged mitochondria for autophagic clearance (42). Growing evidence has demonstrated that impaired PINK1/Parkin mediated-mitophagy contributed significantly to the pathogenesis of DKD, and its enhancement has emerged as a promising therapeutic approach for DKD (43, 44). In this study, we found that inhibition of sEH significantly promoted PINK1/Parkin mediated-mitophagy, alleviated mitochondrial damage, and reduced mtROS generation in HG-stimulated HK-2 cells and diabetic mouse kidneys. Importantly, emerging studies have established mitophagy as a critical regulatory mechanism in the modulation of inflammatory responses (45). Promoting mitophagy attenuated the process of inflammation response and played a protective role in nonalcoholic steatohepatitis (NASH) (46), Alzheimer’s disease (47) and atherogenesis (48). In the current study, we found that inhibition of sEH promoted PINK1/Parkin mediated-mitophagy and attenuated mitochondrial damage and mtROS generation in HG-stimulated HK-2 cells and diabetic mouse kidneys. Moreover, activation of mitophagy with Rapa alleviated mitochondrial damage, mtROS generation and NLRP3 inflammasome activation in HG-stimulated HK-2 cells. This raises the question of whether inhibition of sEH could serve as a therapeutic strategy to intervene in NLRP3 inflammasome activation by regulating mitophagy in DKD. Interestingly, we found that using mitophagy inhibitors 3-MA blunted the inhibitory effect of sEH inhibition on NLRP3 inflammasome activation in HG-treated HK-2 cells, while specific mitochondrial-targeted antioxidant by MitoTEMPO reversed these changes, suggesting that inhibition of sEH prevented renal tubular injury via suppressing mtROS-mediated NLRP3 inflammasome activation through promoting mitophagy in DKD.

Notably, several sEH inhibitors currently under clinical evaluation have exhibited anti-inflammatory properties. EC5026 is an orally active sEH inhibitor that resolves inflammation and alleviates neuropathic pain without the addictive potential of opioids (49). GSK2256294A, a safe and well-tolerated sEH inhibitor, significantly increases serum EETs and exhibits a trend for decreased cerebrospinal fluid inflammatory cytokines in critically ill patients with subarachnoid hemorrhage (SAH) (50). Given the critical role of inflammation in DKD pathogenesis, our mechanistic findings, together with these clinical advancements and the established safety and efficacy profiles of sEH inhibitors, highlight their translational potential and support their further development as an attractive therapeutic strategy for DKD. Meanwhile, further studies are needed to determine whether sEH inhibitors have therapeutic efficacy in models of advanced DKD or in the presence of common comorbid conditions.

This study has several limitations. First, whether inhibition of sEH also suppresses other inflammasomes, such as AIM2 and NLRC4, requires further investigation. Second, although our findings demonstrate that the sEH−mitophagy−inflammasome axis plays an important role in mouse models of DKD, further validation is required to confirm the correlation between sEH expression and mitophagy or inflammasome activation in human DKD samples.

**Figure 10:**
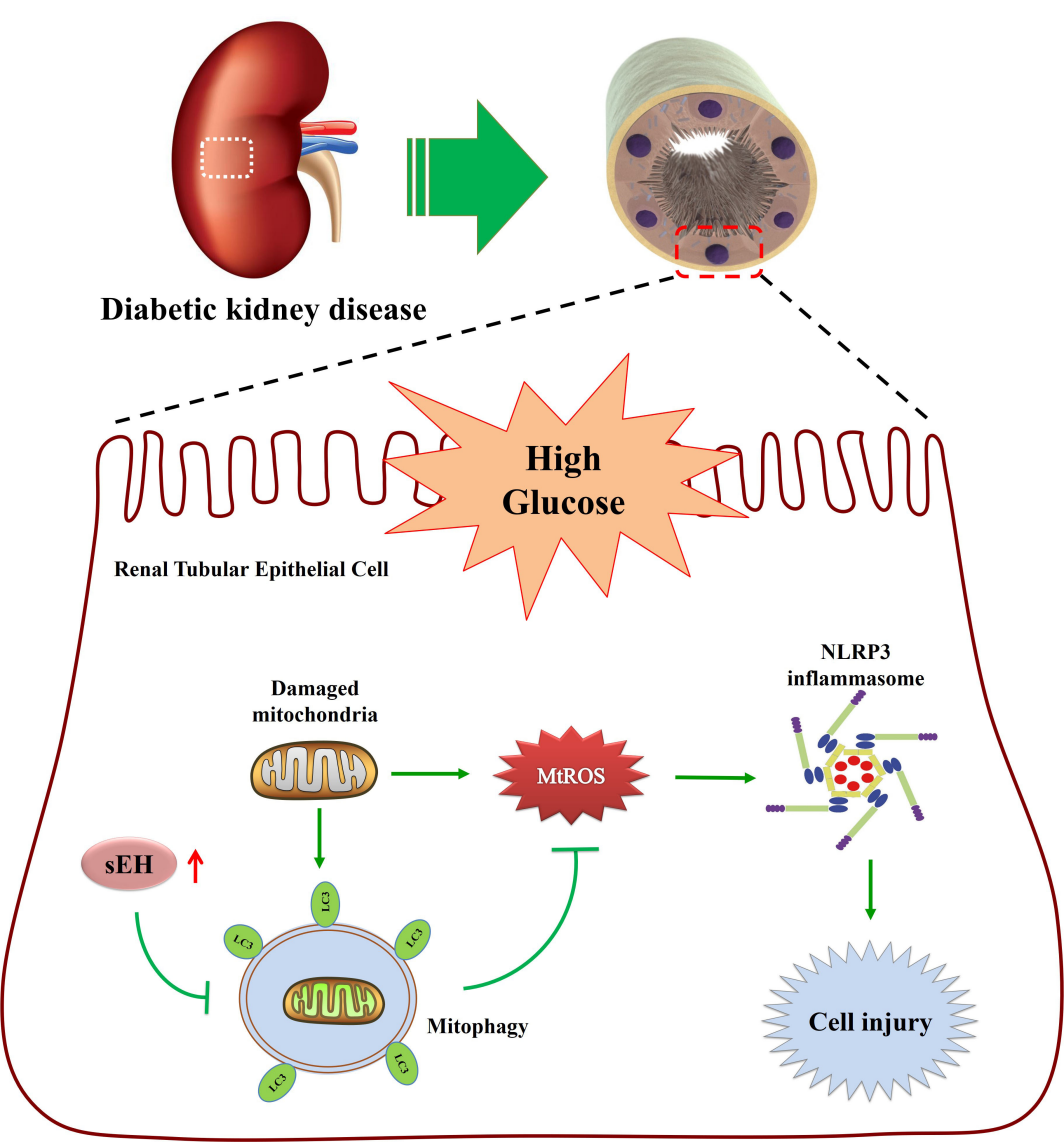
Schematic diagram depicting inhibition of sEH attenuates mtROS-mediated NLRP3 inflammasome activation by promoting mitophagy in tubular epithelial cells in diabetic kidney disease.

In conclusion, our study provided compelling evidence supporting the therapeutic potential of sEH inhibition in attenuating renal tubular inflammatory activation in diabetic kidney disease, as demonstrated in both *in vitro* and *in vivo* models. The protective effects are mediated through a mechanistic pathway wherein sEH inhibition enhanced PINK1/Parkin mediated-mitophagy, reduced mitochondrial ROS generation, and subsequently suppresses NLRP3 inflammasome activation, ultimately preserving renal function in DKD (as summarized in **Figure 10**). These results underscore sEH inhibition as a promising therapeutic strategy for the treatment of DKD.

## Data Availability

The original contributions presented in the study are included in the article/Supplementary Material. Further inquiries can be directed to the corresponding authors.

## Supplementary material

Supplementary Figure 1 Inhibition of sEH enhanced PINK1/Parkin mediated- mitophagy in HG-induced HK-2 cells. (A) Western blot analysis of PINK1 and Parkin protein expression in HK-2 cells after treated with or without *t*-AUCB under HG exposure. (B-C) Densitometric analysis of protein bands from (A). (D) Representative immunofluorescence double-staining images showing the colocalization of Parkin (green) with Mitotracker (red) in HK-2 cells treated with or without *t*-AUCB under HG exposure. (E) Quantification of the colocalization of Parkin and Mitotracker in Figure D. Data are presented as mean ± SEM. (n = 3 independently repeated cultures, **P* < 0.05 vs. Ctr, ^#^*P* < 0.05 vs. *t*-AUCB, ^Δ^*P* < 0.05 vs. HG).

Supplementary Figure 2 Inhibition of sEH promoted mitophagy in the kidneys of T2DM mice. (A) Representative immunofluorescence double-staining images showing the colocalization of VDAC (green) with LC3 (red) in the kidneys of different groups. (B) Representative immunofluorescence double-staining images showing the colocalization of VDAC (green) with Parkin (red) in the kidneys of different groups.

## References

[1] Saeedi P Petersohn I Salpea P Malanda B Karuranga S Unwin N . Global and regional diabetes prevalence estimates for 2019 and projections for 2030 and 2045: Results from the International Diabetes Federation Diabetes Atlas, 9(th) edition. Diabetes Res Clin Pract. (2019) 157:107843. doi: 10.1016/j.diabres.2019.107843. PMID: 31518657

[2] Chevalier RL . The proximal tubule is the primary target of injury and progression of kidney disease: role of the glomerulotubular junction. Am J Physiol Renal Physiol. (2016) 311:F145–61. doi: 10.1152/ajprenal.00164.2016. PMID: 27194714 PMC4967168

[3] Zeni L Norden AGW Cancarini G Unwin RJ . A more tubulocentric view of diabetic kidney disease. J Nephrol. (2017) 30:701–17. doi: 10.1007/s40620-017-0423-9. PMID: 28840540 PMC5698396

[4] Jung CY Yoo TH . Pathophysiologic mechanisms and potential biomarkers in diabetic kidney disease. Diabetes Metab J. (2022) 46:181–97. doi: 10.4093/dmj.2021.0329. PMID: 35385633 PMC8987689

[5] Rayego-Mateos S Rodrigues-Diez RR Fernandez-Fernandez B Mora-Fernández C Marchant V Donate-Correa J . Targeting inflammation to treat diabetic kidney disease: the road to 2030. Kidney Int. (2023) 103:282–96. doi: 10.1016/j.kint.2022.10.030. PMID: 36470394

[6] Mangan MSJ Olhava EJ Roush WR Seidel HM Glick GD Latz E . Targeting the NLRP3 inflammasome in inflammatory diseases. Nat Rev Drug Discov. (2018) 17:588–606. doi: 10.1038/nrd.2018.97. PMID: 30026524

[7] Lin Q Li S Jiang N Jin H Shao X Zhu X . Inhibiting NLRP3 inflammasome attenuates apoptosis in contrast-induced acute kidney injury through the upregulation of HIF1A and BNIP3-mediated mitophagy. Autophagy. (2021) 17:2975–90. doi: 10.1080/15548627.2020.1848971. PMID: 33345685 PMC8525960

[8] Oliveira CB Lima CAD Vajgel G Sandrin-Garcia P . The role of NLRP3 inflammasome in lupus nephritis. Int J Mol Sci. (2021) 22(22):12476. doi: 10.3390/ijms222212476. PMID: 34830358 PMC8625721

[9] Shahzad K Fatima S Khawaja H Elwakiel A Gadi I Ambreen S . Podocyte-specific Nlrp3 inflammasome activation promotes diabetic kidney disease. Kidney Int. (2022) 102:766–79. doi: 10.1016/j.kint.2022.06.010. PMID: 35779608

[10] Jiang XS Liu T Xia YF Gan H Ren W Du XG . Activation of the Nrf2/ARE signaling pathway ameliorates hyperlipidemia-induced renal tubular epithelial cell injury by inhibiting mtROS-mediated NLRP3 inflammasome activation. Front Immunol. (2024) 15:1342350. doi: 10.3389/fimmu.2024.1342350. PMID: 38720901 PMC11076710

[11] Harris TR Hammock BD . Soluble epoxide hydrolase: gene structure, expression and deletion. Gene. (2013) 526:61–74. doi: 10.1016/j.gene.2013.05.008. PMID: 23701967 PMC3733540

[12] He J Wang C Zhu Y Ai D . Soluble epoxide hydrolase: A potential target for metabolic diseases. J Diabetes. (2016) 8:305–13. doi: 10.1111/1753-0407.12358. PMID: 26621325

[13] Wang B Wu L Chen J Dong L Chen C Wen Z . Metabolism pathways of arachidonic acids: mechanisms and potential therapeutic targets. Signal Transduct Target Ther. (2021) 6:94. doi: 10.1038/s41392-020-00443-w. PMID: 33637672 PMC7910446

[14] Sun CP Zhang XY Morisseau C Hwang SH Zhang ZJ Hammock BD . Discovery of soluble epoxide hydrolase inhibitors from chemical synthesis and natural products. J Med Chem. (2021) 64:184–215. doi: 10.1021/acs.jmedchem.0c01507. PMID: 33369424 PMC7942193

[15] Zhou Z Zhang M Zhao C Gao X Wen Z Wu J . Epoxyeicosatrienoic acids prevent cardiac dysfunction in viral myocarditis via interferon type I signaling. Circ Res. (2023) 133:772–88. doi: 10.1161/circresaha.123.322619. PMID: 37681352

[16] Niu Q Guo Z Liang Y Zuo L . Soluble epoxide hydrolase inhibition attenuates proteinuria by alleviating renal inflammation and podocyte injuries in adriamycin-induced nephropathy. Int J Mol Sci. (2024) 25(19):10629. doi: 10.3390/ijms251910629. PMID: 39408958 PMC11476994

[17] Zhang J Zhang M Zhang WH Zhu QM Huo XK Sun CP . Total flavonoids of Inula japonica alleviated the inflammatory response and oxidative stress in LPS-induced acute lung injury via inhibiting the sEH activity: Insights from lipid metabolomics. Phytomedicine. (2022) 107:154380. doi: 10.1016/j.phymed.2022.154380. PMID: 36150346

[18] Jiang XS Xiang XY Chen XM He JL Liu T Gan H . Inhibition of soluble epoxide hydrolase attenuates renal tubular mitochondrial dysfunction and ER stress by restoring autophagic flux in diabetic nephropathy. Cell Death Dis. (2020) 11:385. doi: 10.1038/s41419-020-2594-x. PMID: 32439839 PMC7242354

[19] Wilson PC Wu H Kirita Y Uchimura K Ledru N Rennke HG . The single-cell transcriptomic landscape of early human diabetic nephropathy. Proc Natl Acad Sci USA. (2019) 116:19619–25. doi: 10.1073/pnas.1908706116. PMID: 31506348 PMC6765272

[20] Qin XH Wu Z Dong JH Zeng YN Xiong WC Liu C . Liver soluble epoxide hydrolase regulates behavioral and cellular effects of chronic stress. Cell Rep. (2019) 29:3223–3234.e6. doi: 10.1016/j.celrep.2019.11.006. PMID: 31801085

[21] Tang SCW Yiu WH . Innate immunity in diabetic kidney disease. Nat Rev Nephrol. (2020) 16:206–22. doi: 10.1038/s41581-019-0234-4. PMID: 31942046

[22] Swanson KV Deng M Ting JP . The NLRP3 inflammasome: molecular activation and regulation to therapeutics. Nat Rev Immunol. (2019) 19:477–89. doi: 10.1038/s41577-019-0165-0. PMID: 31036962 PMC7807242

[23] Gupta S Cassel SL Sutterwala FS Dagvadorj J . Regulation of the NLRP3 inflammasome by autophagy and mitophagy. Immunol Rev. (2025) 329:e13410. doi: 10.1111/imr.13410. PMID: 39417249 PMC12477832

[24] Dominic A Le NT Takahashi M . Loop between NLRP3 inflammasome and reactive oxygen species. Antioxid Redox Signal. (2022) 36:784–96. doi: 10.1089/ars.2020.8257. PMID: 34538111

[25] Meier DT de Paula Souza J Donath MY . Targeting the NLRP3 inflammasome-IL-1β pathway in type 2 diabetes and obesity. Diabetologia. (2025) 68:3–16. doi: 10.1007/s00125-024-06306-1. PMID: 39496966 PMC11663173

[26] Danielski LG Giustina AD Bonfante S Barichello T Petronilho F . The NLRP3 inflammasome and its role in sepsis development. Inflammation. (2020) 43:24–31. doi: 10.1007/s10753-019-01124-9. PMID: 31741197

[27] McManus RM Latz E . NLRP3 inflammasome signalling in Alzheimer’s disease. Neuropharmacology. (2024) 252:109941. doi: 10.1016/j.neuropharm.2024.109941. PMID: 38565393

[28] Grebe A Hoss F Latz E . NLRP3 inflammasome and the IL-1 pathway in atherosclerosis. Circ Res. (2018) 122:1722–40. doi: 10.1161/circresaha.118.311362. PMID: 29880500

[29] Luo Y Long M Wu X Zeng L . Targeting the NLRP3 inflammasome in kidney disease: molecular mechanisms, pathogenic roles, and emerging small-molecule therapeutics. Front Immunol. (2025) 16:1703560. doi: 10.3389/fimmu.2025.1703560. PMID: 41357229 PMC12675237

[30] Cliff CL Squires PE Hills CE . Tonabersat suppresses priming/activation of the NOD-like receptor protein-3 (NLRP3) inflammasome and decreases renal tubular epithelial-to-macrophage crosstalk in a model of diabetic kidney disease. Cell Commun Signal. (2024) 22:351. doi: 10.1186/s12964-024-01728-1. PMID: 38970061 PMC11225428

[31] Yu JW Lee MS . Mitochondria and the NLRP3 inflammasome: physiological and pathological relevance. Arch Pharm Res. (2016) 39:1503–18. doi: 10.1007/s12272-016-0827-4. PMID: 27600432

[32] Subramanian N Natarajan K Clatworthy MR Wang Z Germain RN . The adaptor MAVS promotes NLRP3 mitochondrial localization and inflammasome activation. Cell. (2013) 153:348–61. doi: 10.1016/j.cell.2013.02.054. PMID: 23582325 PMC3632354

[33] Ichinohe T Yamazaki T Koshiba T Yanagi Y . Mitochondrial protein mitofusin 2 is required for NLRP3 inflammasome activation after RNA virus infection. Proc Natl Acad Sci USA. (2013) 110:17963–8. doi: 10.1073/pnas.1312571110. PMID: 24127597 PMC3816452

[34] Han Y Xu X Tang C Gao P Chen X Xiong X . Reactive oxygen species promote tubular injury in diabetic nephropathy: The role of the mitochondrial ros-txnip-nlrp3 biological axis. Redox Biol. (2018) 16:32–46. doi: 10.1016/j.redox.2018.02.013. PMID: 29475133 PMC5842313

[35] Zhang J Tu M Liu Z Zhang G . Soluble epoxide hydrolase as a therapeutic target for obesity-induced disorders: roles of gut barrier function involved. Prostaglandins Leukot Essent Fatty Acids. (2020) 162:102180. doi: 10.1016/j.plefa.2020.102180. PMID: 33038829 PMC7669660

[36] Wang YX Ulu A Zhang LN Hammock B . Soluble epoxide hydrolase in atherosclerosis. Curr Atheroscler Rep. (2010) 12:174–83. doi: 10.1007/s11883-010-0108-5. PMID: 20425256 PMC2857794

[37] Huang H Weng J Wang MH . EETs/sEH in diabetes and obesity-induced cardiovascular diseases. Prostaglandins Other Lipid Mediat. (2016) 125:80–9. doi: 10.1016/j.prostaglandins.2016.05.004. PMID: 27184755

[38] Zhang CY Tan XH Yang HH Jin L Hong JR Zhou Y . COX-2/sEH dual inhibitor alleviates hepatocyte senescence in NAFLD mice by restoring autophagy through Sirt1/PI3K/AKT/mTOR. Int J Mol Sci. (2022) 23(15):8267. doi: 10.3390/ijms23158267. PMID: 35897843 PMC9332821

[39] Yang HH Duan JX Liu SK Xiong JB Guan XX Zhong WJ . A COX-2/sEH dual inhibitor PTUPB alleviates lipopolysaccharide-induced acute lung injury in mice by inhibiting NLRP3 inflammasome activation. Theranostics. (2020) 10:4749–61. doi: 10.7150/thno.43108. PMID: 32308747 PMC7163435

[40] Sun CC Zhang CY Duan JX Guan XX Yang HH Jiang HL . PTUPB ameliorates high-fat diet-induced non-alcoholic fatty liver disease via inhibiting NLRP3 inflammasome activation in mice. Biochem Biophys Res Commun. (2020) 523:1020–6. doi: 10.1016/j.bbrc.2019.12.131. PMID: 31973813 PMC7990110

[41] Picca A Faitg J Auwerx J Ferrucci L D'Amico D . Mitophagy in human health, ageing and disease. Nat Metab. (2023) 5:2047–61. doi: 10.1038/s42255-023-00930-8. PMID: 38036770 PMC12159423

[42] Narendra DP Youle RJ . The role of PINK1-Parkin in mitochondrial quality control. Nat Cell Biol. (2024) 26:1639–51. doi: 10.1038/s41556-024-01513-9. PMID: 39358449

[43] Xiao L Xu X Zhang F Wang M Xu Y Tang D . The mitochondria-targeted antioxidant MitoQ ameliorated tubular injury mediated by mitophagy in diabetic kidney disease via Nrf2/PINK1. Redox Biol. (2017) 11:297–311. doi: 10.1016/j.redox.2016.12.022. PMID: 28033563 PMC5196243

[44] Wang YH Chang DY Zhao YY Tang SC Zhao MH Chen M . Mitochondrial protein TOMM7 alleviates diabetic kidney disease by regulating mitophagy via intracellular redistribution of phospholipase PLA2G6. Kidney Int. (2026) 109:337–53. doi: 10.1016/j.kint.2025.10.009. PMID: 41276015

[45] Zhao Y Huang S Liu J Wu X Zhou S Dai K . Mitophagy contributes to the pathogenesis of inflammatory diseases. Inflammation. (2018) 41:1590–600. doi: 10.1007/s10753-018-0835-2. PMID: 29959626

[46] Lv T Fan X He C Zhu S Xiong X Yan W . SLC7A11-ROS/αKG-AMPK axis regulates liver inflammation through mitophagy and impairs liver fibrosis and NASH progression. Redox Biol. (2024) 72:103159. doi: 10.1016/j.redox.2024.103159. PMID: 38642501 PMC11047786

[47] Guo M Wang Z Zhou X Yu C Wu J Yu L . Ciliatoside A attenuates neuroinflammation in Alzheimer’s disease by activating mitophagy and inhibiting NLRP3 inflammasome activation. Phytomedicine. (2025) 145:156928. doi: 10.1016/j.phymed.2025.156928. PMID: 40541122

[48] Hu Q Li C Zhang T Yi L Shan Y Ma X . Dihydromyricetin suppresses endothelial NLRP3 inflammasome activation and attenuates atherogenesis by promoting mitophagy. Lipids Health Dis. (2024) 23:279. doi: 10.1186/s12944-024-02263-1. PMID: 39227809 PMC11370113

[49] Hammock BD McReynolds CB Wagner K Buckpitt A Cortes-Puch I Croston G . Movement to the clinic of soluble epoxide hydrolase inhibitor EC5026 as an analgesic for neuropathic pain and for use as a nonaddictive opioid alternative. J Med Chem. (2021) 64:1856–72. doi: 10.1021/acs.jmedchem.0c01886. PMID: 33550801 PMC7917437

[50] Martini RP Siler D Cetas J Alkayed NJ Allen E Treggiari MM . A double-blind, randomized, placebo-controlled trial of soluble epoxide hydrolase inhibition in patients with aneurysmal subarachnoid hemorrhage. Neurocrit Care. (2022) 36:905–15. doi: 10.1007/s12028-021-01398-8. PMID: 34873674

