# Supplementary figures and images for: sEH inhibition attenuates mtROS-mediated NLRP3 inflammasome activation by promoting mitophagy in tubular epithelial cells in diabetic kidney disease
Source: Front Immunol. 2026 Apr 28;17:1767802. doi: 10.3389/fimmu.2026.1767802 (PMC13160782; doi:10.3389/fimmu.2026.1767802)

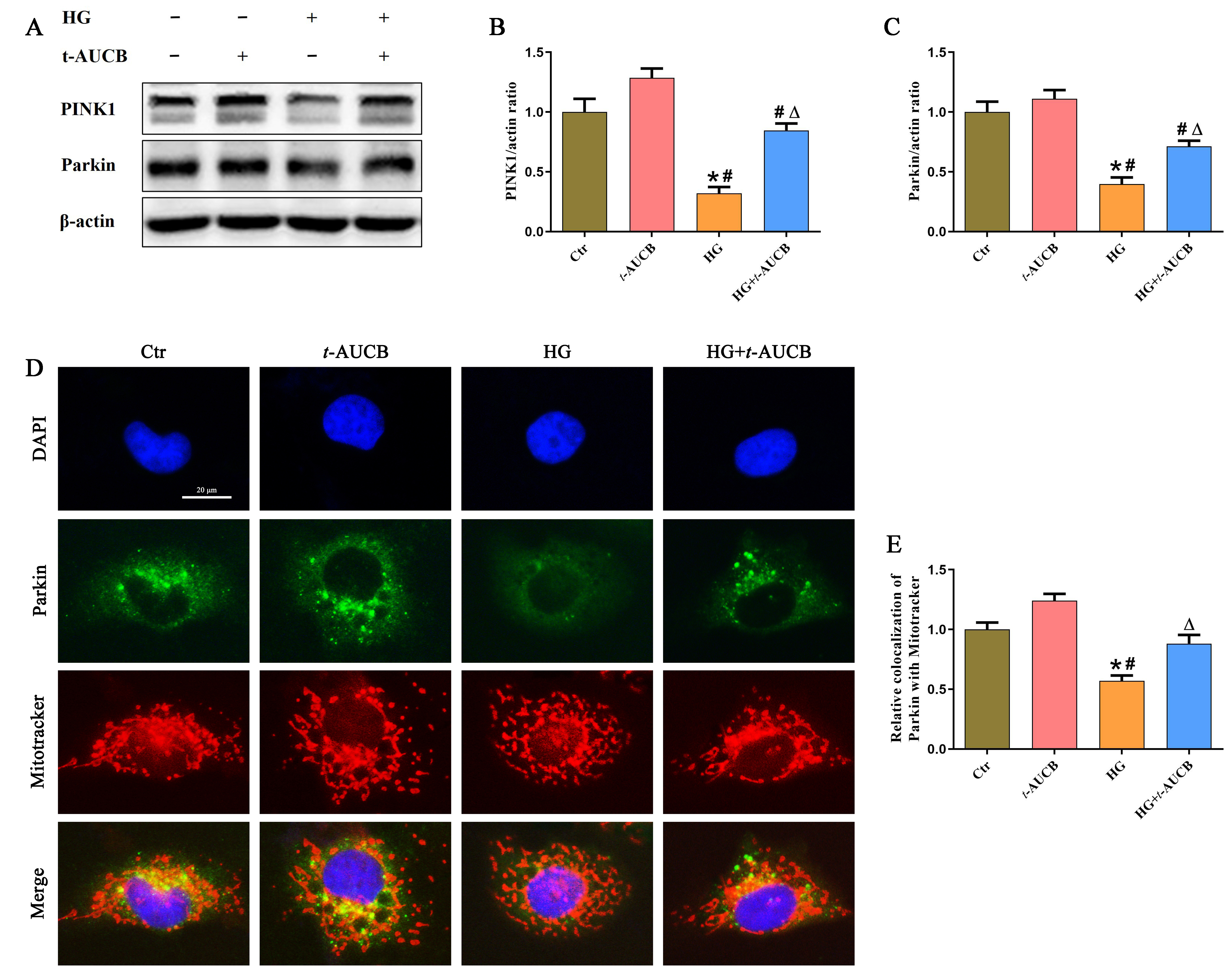

Supplement: Supplementary Figure 1 — Inhibition of sEH enhanced PINK1/Parkin mediated- mitophagy in HG-induced HK-2 cells. (A) Western blot analysis of PINK1 and Parkin protein expression in HK-2 cells after treated with or without t-AUCB under HG exposure. (B-C) Densitometric analysis of protein bands from (A). (D) Representative immunofluorescence double-staining images showing the colocalization of Parkin (green) with Mitotracker (red) in HK-2 cells treated with or without t-AUCB under HG exposure. (E) Quantification of the colocalization of Parkin and Mitotracker in Figure D. Data are presented as mean ± SEM. (n = 3 independently repeated cultures, *P < 0.05 vs. Ctr, #P < 0.05 vs. t-AUCB, ΔP < 0.05 vs. HG). [file Image1.jpeg]

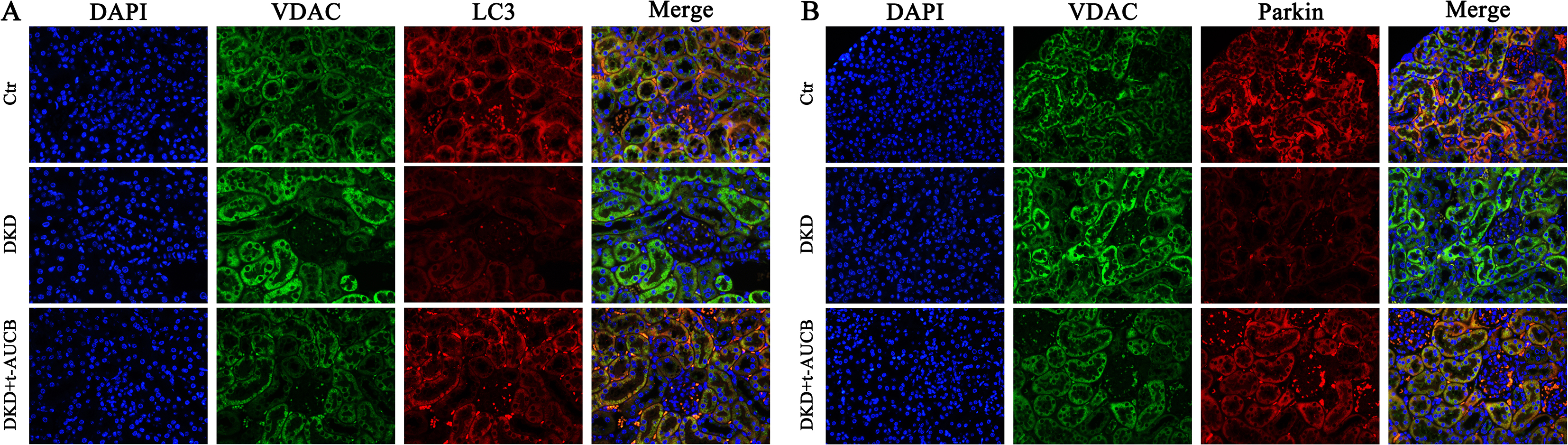

Supplement: Supplementary Figure 2 — Inhibition of sEH promoted mitophagy in the kidneys of T2DM mice. (A) Representative immunofluorescence double-staining images showing the colocalization of VDAC (green) with LC3 (red) in the kidneys of different groups. (B) Representative immunofluorescence double-staining images showing the colocalization of VDAC (green) with Parkin (red) in the kidneys of different groups. [file Image2.jpeg]
